# Clinical Impact of Switching From or Persisting With Rivaroxaban or Apixaban After a Bleeding Event: A Real‐World Study in Patients With Nonvalvular Atrial Fibrillation

**DOI:** 10.1161/JAHA.125.044113

**Published:** 2026-01-19

**Authors:** Gregory Y. H. Lip, Dong Cheng, Jenny Jiang, Chuan Gao, Rupesh Subash, Elisabeth Vodicka, Anandkumar Dubey, Puza P. Sharma, Anakha Vettumthara Unnikrishnan, Kishorekumar Mathivanan, Steven Deitelzweig

**Affiliations:** ^1^ Liverpool Centre for Cardiovascular Science at University of Liverpool Liverpool John Moores University and Liverpool Heart & Chest Hospital Liverpool United Kingdom; ^2^ Danish Center for Health Services Research, Department of Clinical Medicine Aalborg University Aalborg Denmark; ^3^ Department of Cardiology, Lipidology and Internal Medicine with Intensive Coronary Care Unit Medical University of Bialystok Bialystok Poland; ^4^ Bristol Myers Squibb Company Lawrenceville NJ USA; ^5^ Pfizer Ltd. Tadworth United Kingdom; ^6^ Pfizer Inc. New York NY USA; ^7^ Mu Sigma Business Solutions Pvt. Ltd Bangalore India; ^8^ Ochsner Clinic Foundation, Department of Hospital Medicine New Orleans LA USA; ^9^ University of Queensland School of Medicine—Ochsner Clinical School New Orleans LA USA

**Keywords:** atrial fibrillation, direct oral anticoagulant, hemorrhage, stroke, switching, Anticoagulants, Atrial Fibrillation

## Abstract

**Background:**

Real‐world research has identified differences in effectiveness and safety between direct oral anticoagulants (DOACs) when used to reduce risks of stroke and systemic embolism in patients with atrial fibrillation. However, treatment decisions and subsequent outcomes are poorly understood in patients with atrial fibrillation who experience bleeding when receiving a DOAC.

**Methods:**

A retrospective observational study was conducted using US claims data from Optum’s Clinformatics Data Mart Database (January 2012 through March 2024). The study population comprised adults with nonvalvular atrial fibrillation who initiated a DOAC and had a subsequent claim for bleeding. After propensity score matching, patients who persisted with the same DOAC after bleeding were compared with those who switched to another DOAC. The main outcomes were major bleeding and stroke/systemic embolism.

**Results:**

Among 59 620 apixaban initiators and 21 004 rivaroxaban initiators with bleeding while on therapy, 408 apixaban‐to‐rivaroxaban switchers were matched with 2040 persistent apixaban users, and 901 rivaroxaban‐to‐apixaban switchers were matched with 4505 persistent rivaroxaban users. Compared with persisting with apixaban following a bleeding event, apixaban‐to‐rivaroxaban switching was associated with a higher risk of major bleeding (hazard ratio [HR], 1.690 [95% CI, 1.130–2.527]) and a similar risk of stroke/systemic embolism (HR, 1.793 [95% CI, 0.765–4.202]). Compared with persisting with rivaroxaban, rivaroxaban‐to‐apixaban switching was associated with a lower risk of major bleeding (HR, 0.651 [95% CI, 0.460–0.920]) and a similar risk of stroke/systemic embolism (HR, 0.783 [95% CI, 0.371–1.652]).

**Conclusions:**

These findings suggest that the choice of DOAC after a bleeding event can affect treatment outcomes in patients with atrial fibrillation.

**Nonstandard Abbreviations and Acronyms**
CCI: Charlson Comorbidity Index
DOAC: direct oral anticoagulant
ICH: intracranial hemorrhage
NVAF: nonvalvular atrial fibrillation
OAC: oral anticoagulant
CDM: Clinformatics Data Mart Database
PSM: propensity score matching
SE: systemic embolism

**Clinical Perspective**

**What Is New?**

- Among patients with nonvalvular atrial fibrillation who experienced a bleeding event, switching from apixaban to rivaroxaban was associated with a higher risk of major bleeding and a similar risk of stroke/systemic embolism, compared with persisting with apixaban.
- Compared with persisting with rivaroxaban, rivaroxaban‐to‐apixaban switching was associated with a lower risk of major bleeding and a similar risk of stroke/systemic embolism.

**What Are the Clinical Implications?**

- The findings from our study may provide useful insights for physicians on direct oral anticoagulant selection after a bleeding event.

Population aging has been accompanied by an increasing incidence of atrial fibrillation (AF), a trend that is anticipated to continue well into the future,^1^, ^2^, ^3^ leading to a major public health burden in terms of health care costs and resources. In the United States (US), deaths related to AF are increasing.^4^ Beyond death, patients with AF experience a major burden from both AF itself and the various cardiovascular and other comorbidities they often have, leading to clinically complex patient phenotypes.^5^, ^6^

AF is associated with an increased risk of stroke, and patients with AF are recommended to receive oral anticoagulant (OAC) treatment to reduce this risk.^7^ For patients with nonvalvular AF (NVAF), a common form of AF not linked to heart valve problems, direct oral anticoagulants (DOACs) such as apixaban, dabigatran, edoxaban, and rivaroxaban are now standard‐of‐care therapy for preventing stroke and systemic embolism (SE) in guidelines globally.^8^, ^9^, ^10^, ^11^ However, anticoagulation in patients with AF is associated with an increased risk of bleeding,^12^ which is a major health burden to patients.^13^, ^14^

Current AF treatment guidelines recommend interrupting OAC treatment while anticoagulation‐associated active bleeding is managed. Although OAC therapy may be resumed once the causes of bleeding have been managed, the guidelines do not include clear guidance on the timing of OAC resumption. Many patients with AF who interrupt anticoagulant treatment after experiencing bleeding do not subsequently restart anticoagulation,^13^, ^15^, ^16^ which puts them at greater risk of stroke and death.^16^, ^17^ Moreover, the reasons why patients do not restart OAC treatment after a bleeding event are poorly understood.

Current treatment guidelines also provide little clarity on OAC treatment selection after a bleeding event, including whether to resume the original OAC or switch to another OAC. Real‐world data show differences in effectiveness and safety between different DOACs when administered in the first‐line setting to patients with AF^18^, ^19^, ^20^, ^21^ but did not examine treatment decisions and outcomes subsequent to an initial bleeding event. A previous retrospective analysis of claims data for US patients with NVAF found that, compared with continuing on the same DOAC, switching from apixaban to rivaroxaban was associated with higher risks of major bleeding and stroke/SE, while switching from rivaroxaban to apixaban was associated with a lower risk of major bleeding and a similar risk of stroke/SE.^22^ However, patients who switched their DOAC for medical reasons were not distinguished from those who switched for nonmedical reasons, so the impact of DOAC switching specifically for medical reasons could not be discerned.

To extend this previous study^22^ and establish outcomes of DOAC switching for medical reasons, DOAC treatment patterns and baseline characteristics of patients with NVAF who experienced a bleeding event were analyzed, and rates of major bleeding and stroke/SE were compared between those who switched their DOAC following the bleeding event and those who persisted with the same DOAC.

## METHODS

Restrictions apply to the availability of the data that support the findings of this study. The data sets generated and analyzed for the current study are not publicly available due to the confidential and proprietary nature of the data sets.

### Study Design and Data Source

This was a retrospective observational study performed using US claims data from Optum’s deidentified Clinformatics Data Mart Database (Optum CDM), which is derived from a database of administrative health claims for members of large commercial and Medicare Advantage health plans.^23^ Optum CDM uses medical and pharmacy claims to derive patient‐level enrollment information, health care costs, and resource use information. The population is geographically diverse, spanning all 50 states, and is statistically deidentified under the Health Insurance Portability and Accountability Act Privacy Rule’s Expert Determination method and managed according to Optum customer data use agreements. Optum CDM administrative claims submitted for payment by providers and pharmacies are verified, adjudicated, and deidentified before inclusion. Access to the data in Optum CDM was not limited in any way. Data were extracted for the study period of January 1, 2012, through March 31, 2024. The application of data cleaning methods was not necessary. Ethics committee approval and patient consent were not required because anonymized aggregated data were used and identifiable personal information was not collected.

### Patient Selection

The study population comprised adults (aged ≥18 years) with NVAF who initiated an OAC during the OAC initiation period: January 1, 2013, through September 30, 2023. National Drug Codes were used to identify OACs (Table S1). Patients whose first OAC claim was for warfarin were excluded, meaning that the sample comprised DOAC initiators.

Included patients had at least 1 claim with an *International Classification of Diseases* (*ICD*) diagnosis code for AF (*ICD*, *Ninth Revision, Clinical Modification* [*ICD‐9‐CM*] 427.31; *ICD*, *Tenth Revision, Clinical Modification* [*ICD‐10‐CM*] I480, I481, I482, or I4891; recorded as a primary or secondary diagnosis in an inpatient or outpatient setting) on or before the index DOAC initiation date: the prescription fill date for their first DOAC claim during the OAC initiation period. They also had ≥6 months of continuous enrollment before the DOAC initiation date. The first DOAC they initiated was designated the index DOAC.

Included patients further had at least 1 outpatient or inpatient claim for a bleeding event (diagnosis or procedure in any position) in the interval from the index DOAC initiation date until 30 days after the end of the period covered by the last filled prescription for their index DOAC. The first bleeding event in this period was designated the index bleeding event. *ICD* codes used to identify index bleeding events are listed in Table S2.

Patients were excluded if they had valvular heart disease or heart valve replacement or transplant in the 6 months before or on the DOAC initiation date; had venous thromboembolism or transient AF in the 6 months before or on the DOAC initiation date; underwent hip or knee replacement surgery in the 6 weeks before or on the DOAC initiation date; were pregnant anytime during the study period; were prescribed an OAC in the 6 months before the DOAC initiation date; had >1 OAC prescription claim on the DOAC initiation date; were receiving multiple OACs at the time of the index bleeding event; or had 0 days of follow‐up.

### Patient Cohorts and Follow‐Up

**Figure 1:**
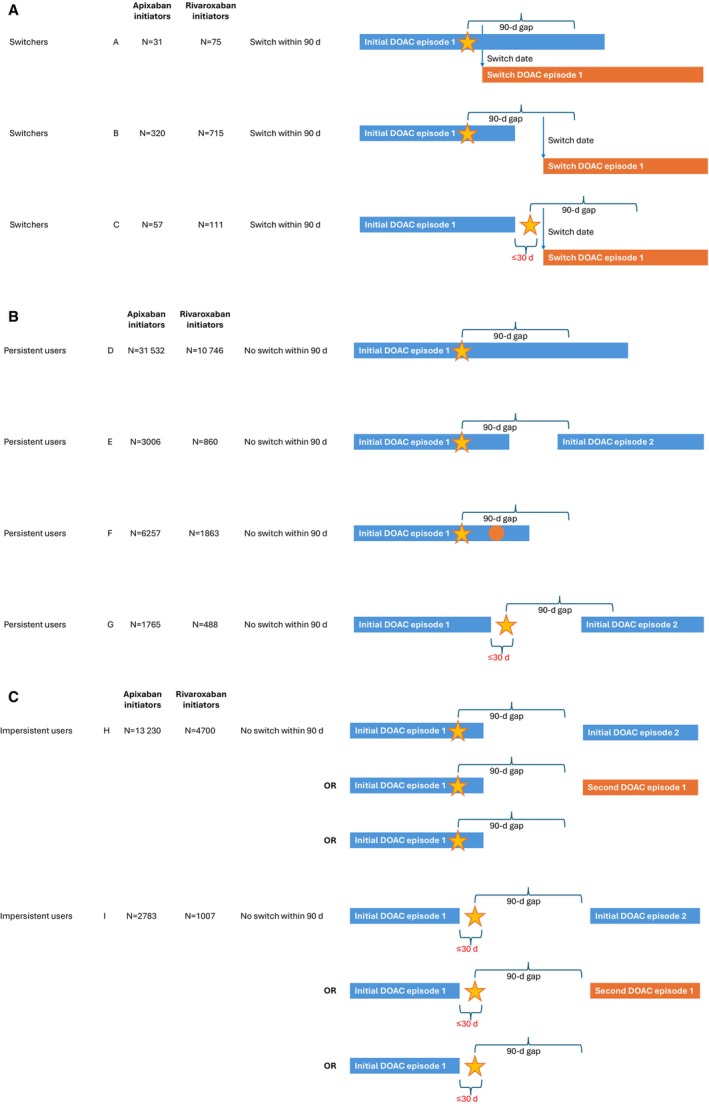
Makeup of the different cohorts. **A**, Switchers. **B**, Persistent users. **C**, Impersistent users. Stars indicate index bleeding events. Blue arrows indicate switch dates. The orange circle indicates a refill after the index bleeding event. In defining an episode of DOAC use, a grace period of up to 30 days between consecutive prescriptions of the same DOAC was allowed. For switchers, the first claim for the DOAC to which they switched had to be within 90 days of the index bleeding event. Persistent DOAC users remained on their index DOAC from the DOAC initiation date until ≥90 days after the index bleeding event, reinitiated their index DOAC within 90 days after the index bleeding event, or had a refill of their index DOAC after the index bleeding event. Impersistent users did not switch their DOAC or reinitiate their index DOAC within 90 days after the index bleeding event. DOAC indicates direct oral anticoagulant.

Included patients were categorized into switcher, persistent, and impersistent cohorts (Figure 1). Switchers were patients with a claim for a different OAC than the index DOAC within 90 days of the index bleeding event. For switchers, the index date was defined as the switch date: the date of their first claim for the DOAC to which they switched.

Persistent DOAC users remained on their index DOAC therapy from the DOAC initiation date until ≥90 days after the index bleeding event, reinitiated their index DOAC therapy within 90 days after the index bleeding event, or had a refill of their index DOAC after the index bleeding event. For persistent DOAC users, the index date could not be the switch date, since they did not switch OAC (ie, there was no switch date). Instead, pseudo‐switch dates were used as the index date. Pseudo‐switch dates were hypothetical switch dates derived by repeatedly sampling switching times (intervals from the date of the index bleeding event to the switch date) from the switcher cohort and randomly assigning them to the persistent user cohort in the time period from the index bleeding event to the end of follow‐up.

Impersistent DOAC users were patients not included in the switcher or persistent user cohort and included those who did not resume DOAC treatment within 90 days after a bleeding event. For these patients, the index date was the date of the index bleeding event.

In comparisons of the persistent user and switcher cohorts, patient follow‐up ran from the day after the index date to censoring at the earliest of the following: 30 days after the end of treatment (where treatment was specified as the index DOAC for persistent users and the switched DOAC for switchers); switch to another OAC; the end of continuous enrollment; the end of the study period; death; and the first outcome of interest (major bleeding or stroke/SE event; see below). In defining episodes of DOAC use, we calculated the duration of supply on the basis of the number of prescriptions dispensed, the recorded number of days’ supply, and dosing information derived from the National Drug Code associated with each prescription record in Optum CDM. A gap of up to 30 days between prescriptions for the same DOAC was permitted without it being considered a new episode.

### Outcomes

Demographics and baseline clinical characteristics were assessed in the 6‐month period up to and including the index date. They included age (assessed on the index date), selected comorbidities, Charlson Comorbidity Index (CCI),^24^ CHA_2_DS_2_‐VASc score,^25^ and HAS‐BLED score.^26^

Major bleeding and stroke/SE events during follow‐up were identified from inpatient claims in which major bleeding or stroke/SE represented the primary *ICD* diagnosis or procedure. Major bleeding was the primary outcome variable and comprised gastrointestinal bleeding, intracranial hemorrhage (ICH), and major bleeding at other sites. Stroke/SE was a secondary outcome variable and comprised ischemic stroke, hemorrhagic stroke, and SE. The *ICD* codes used to identify major bleeding and stroke/SE are listed in Table S3.

### Statistical Analysis

Summary statistics (mean±SD, median [interquartile range] for continuous variables; n [%] for categorical variables) were calculated for demographics and baseline clinical characteristics.

Power calculations were performed for the primary outcome variable (major bleeding) to determine whether to proceed with a comparative analysis of switchers versus persistent users. The aim was to ensure that any comparative analyses would have sufficient statistical power to detect statistical differences. Further details are provided in Data S1.

To ensure comparability of the switcher and persistent user cohorts, propensity score matching (PSM) with a 1:5 switcher‐to‐persistent user ratio was performed on the basis of baseline characteristics, time from the DOAC initiation date to the index bleeding event, and time from the index bleeding event date to the switch/pseudo‐switch date. PSM used nearest neighbor matching without replacement and a caliper of 0.1. After PSM, baseline characteristics and key study time intervals were compared between switchers and persistent users by calculating the standardized mean difference (SMD) as |actual standard difference|×100. An SMD value >10 was considered statistically significant.

For the post‐PSM switcher and persistent user cohorts, incidence rates per 100 person‐years were calculated for major bleeding and stroke/SE events, and cumulative incidence curves were derived from the Kaplan–Meier survival probabilities (1 minus survival probability at any given time). Hazard ratios (HRs) for major bleeding and stroke/SE in post‐PSM switchers versus persistent users were calculated using Cox proportional hazards models. The proportional hazards assumption was tested by the Schoenfeld residuals method.^27^

### Sensitivity Analyses

Additional Cox models adjusted for variables that were unbalanced after PSM were computed as a sensitivity analysis. In a second sensitivity analysis, HRs were calculated using Fine–Gray models that included death as a competing risk.

To assess the potential influence of unmeasured confounding, we calculated the E‐value as the minimum strength of association an unmeasured confounder would need to have with both the exposure and outcome (conditional on other measured covariates in the model) to fully explain away the observed exposure–outcome association (ie, reduce it to the null).^28^, ^29^

## RESULTS

### Study Population

**Figure 2:**
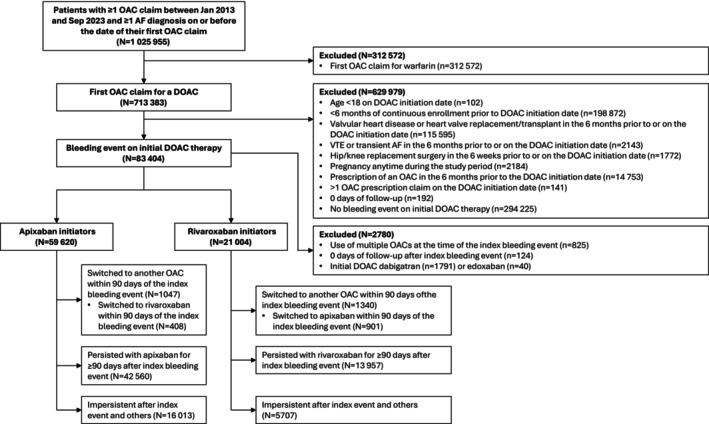
Patient disposition. AF indicates atrial fibrillation; DOAC, direct oral anticoagulant; OAC, oral anticoagulant; and VTE, venous thromboembolism.

In total, 1 025 955 patients with at least 1 OAC claim and at least 1 AF diagnosis on or before the date of their first OAC claim were identified between January 2013 and September 2023 (Figure 2). Applying the exclusion criteria yielded 83 404 patients with a bleeding event on their initial DOAC and ultimately resulted in a sample that included 59 620 apixaban initiators and 21 004 rivaroxaban initiators, as well as 1791 dabigatran initiators and 40 edoxaban initiators. Because of their small sample sizes, dabigatran and edoxaban initiators were excluded from the present analysis, which thus focused solely on apixaban and rivaroxaban.

Of the apixaban initiators, 42 560 (71.4%) were persistent apixaban users, 16 013 (26.9%) were impersistent users, and 1047 (1.8%) switched to another OAC, with 408 apixaban initiators (0.7%) specifically switching to rivaroxaban. For rivaroxaban initiators, 13 957 (66.5%) were persistent rivaroxaban users, 5707 (27.2%) were impersistent users, and 1340 (6.4%) switched to another OAC, with 901 rivaroxaban initiators (4.3%) specifically switching to apixaban.

### Patient Characteristics Before PSM

Overall, apixaban initiators who experienced bleeding while on therapy had a greater disease burden at baseline than rivaroxaban initiators who experienced bleeding, with a higher mean CCI score (4.01 versus 3.21), a higher mean CHA_2_DS_2_‐VASc score (4.61 versus 4.15), a higher mean HAS‐BLED score (3.95 versus 3.66), and higher frequencies of multiple comorbidities and all‐cause hospitalization (Table S4). Compared with rivaroxaban initiators, apixaban initiators were also slightly older (mean age, 76.16 versus 74.21 years).

### Apixaban Initiators

Table 1 shows pre‐PSM baseline characteristics for apixaban‐to‐rivaroxaban switchers and persistent and impersistent apixaban users. For over 85% of apixaban switchers, persistent users, and impersistent users, the index bleeding event was clinically relevant nonmajor bleeding. Most index bleeding events (>60% for apixaban switchers and persistent users, 54% for impersistent apixaban users) were of individual types (ie, not involving multiple sites/types) other than ICH and gastrointestinal bleeding. Compared with persistent apixaban users, greater proportions of impersistent users had ICH or multiple types of bleeding as the index bleeding event.

**Table 1:** Pre‐PSM Baseline Characteristics of Switchers, Persistent Users, and Impersistent Users

	Apixaban initiators			Rivaroxaban initiators		
	Apixaban‐to‐rivaroxaban switchers (N=408)	Persistent apixaban users (N=42 560)	Impersistent apixaban users (n=16 013)	Rivaroxaban‐to‐apixaban switchers (N=901)	Persistent rivaroxaban users (N=13 957)	Impersistent rivaroxaban users (N=5707)
Age, y						
Mean±SD	75.43±8.16	75.93±8.72	76.91±8.87	74.92±9.30	73.88±9.48	75.04±9.58
Median (IQR)	76 (70–81.5)	77 (70–83)	78 (71–84)	76 (70–82)	75 (68–81)	76 (69–82)
Age category, y, n (%)						
18–54	5 (1.23)	760 (1.79)	286 (1.79)	31 (3.44)	481 (3.45)	176 (3.08)
55–64	31 (7.60)	2853 (6.70)	936 (5.85)	69 (7.66)	1390 (9.96)	525 (9.20)
65–74	125 (30.64)	12 332 (28.98)	4349 (27.16)	271 (30.08)	4521 (32.39)	1710 (29.96)
75–79	94 (23.04)	8951 (21.03)	3207 (20.03)	198 (21.98)	2762 (19.79)	1107 (19.40)
≥80	153 (37.50)	17 664 (41.50)	7235 (45.18)	332 (36.85)	4803 (34.41)	2189 (38.36)
Sex, n (%)						
Female	213 (52.21)	21 162 (49.72)	7580 (47.34)	419 (46.50)	6228 (44.62)	2495 (43.72)
Male	195 (47.79)	21 391 (50.26)	8431 (52.65)	482 (53.50)	7726 (55.36)	3209 (56.23)
Unknown	0 (0.00)	7 (0.02)	<5*	0 (0.00)	<5*	<5*
Site/type of index bleeding event, n (%)						
Gastrointestinal	90 (22.06)	9368 (22.01)	3489 (21.79)	197 (21.86)	2915 (20.89)	1271 (22.27)
Intracranial hemorrhage	28 (6.86)	1609 (3.78)	1383 (8.64)	29 (3.22)	355 (2.54)	357 (6.26)
Other	259 (63.48)	29 182 (68.57)	8619 (53.83)	528 (58.60)%	9962 (71.38)	3086 (54.07)
Multiple	31 (7.60)	2401 (5.64)	2522 (15.75)	147 (16.32)	725 (5.19)	993 (17.40)
Severity of index bleeding event, n (%)						
Major bleeding	26 (6.37)	1566 (3.68)	1952 (12.19)	119 (13.21)	520 (3.73)	824 (14.44)
Clinically relevant nonmajor bleeding	382 (93.63)	40 994 (96.32)	14 061 (87.81)	782 (86.79)	13 437 (96.27)	4883 (85.56)
CCI score						
Mean±SD	4.26±2.87	3.83±2.86	4.87±3.08	4.11±2.93	3.01±2.54	3.89±2.88
Median (IQR)	4 (2–6)	3 (2–6)	5 (2–7)	4 (2–6)	2 (1–4)	3 (2–6)
CHA_2_DS_2_‐VASc score						
Mean±SD	4.82±1.72	4.57±1.69	4.88±1.69	4.64±1.70	4.06±1.68	4.41±1.74
Median (IQR)	5 (4–6)	5 (3–6)	5 (4–6)	5 (4–6)	4 (3–5)	4 (3–6)
CHA_2_DS_2_‐VASc score, n (%)						
0	<5*	150 (0.35)	47 (0.29)	<5*	111 (0.80)	46 (0.81)
1	8 (1.96)	917 (2.15)	286 (1.79)	25 (2.77)	604 (4.33)	224 (3.93)
2	26 (6.37)	3513 (8.25)	882 (5.51)	68 (7.55)	1749 (12.53)	494 (8.66)
3	53 (12.99)	6889 (16.19)	2086 (13.03)	127 (14.10)	2926 (20.96)	968 (16.96)
≥4	319 (78.19)	31 091 (73.05)	12 712 (79.39)	677 (75.14)	8567 (61.38)	3975 (69.65)
HAS‐BLED score						
Mean±SD	4.09±1.14	3.90±1.09	4.18±1.11	3.99±1.10	3.59±1.07	3.86±1.11
Median (IQR)	4 (3–5)	4 (3–5)	4 (3–5)	4 (3–5)	4 (3–4)	4 (3–5)
HAS‐BLED score, n (%)						
0	0 (0.00)	0 (0.00)	0 (0.00)	0 (0.00)	0 (0.00)	0 (0.00)
1	<5*	280 (0.66)	84 (0.52)	9 (1.00)	186 (1.33)	84 (1.47)
2	22 (5.39)	3231 (7.59)	747 (4.66)	52 (5.77)	1710 (12.25)	476 (8.34)
≥3	383 (93.87)	39 049 (91.75)	15 182 (94.81)	840 (93.23)	12 061 (86.42)	5147 (90.19)
Baseline comorbidities, n (%)						
History of any bleeding	408 (100.00)	42 560 (100.00)	16 013 (100.00)	901 (100.00)	13 957 (100.00)	5707 (100.00)
Congestive heart failure	184 (45.10)	17 066 (40.10)	7998 (49.95)	394 (43.73)	4455 (31.92)	2341 (41.02)
Diabetes	169 (41.42)	16 724 (39.30)	7101 (44.35)	352 (39.07)	4908 (35.17)	2265 (39.69)
Hypertension	377 (92.40)	38 571 (90.63)	14 892 (93.00)	844 (93.67)	12 252(87.78)	5146 (90.17)
Renal disease	177 (43.38)	18 350 (43.12)	9108 (56.88)	417 (46.28)	4225 (30.27)	2342 (41.04)
Liver disease	54 (13.24)	4079 (9.58)	2207 (13.78)	112 (12.43)	1137 (8.15)	624 (10.93)
Myocardial infarction	73 (17.89)	7099 (16.68)	3429 (21.41)	162 (17.98)	1733 (12.42)	978 (17.14)
Dyspepsia or stomach discomfort	109 (26.72)	9571 (22.49)	4238 (26.47)	188 (20.87)	2266 (16.24)	1049 (18.38)
Peripheral vascular disease	158 (38.73)	14 533 (34.15)	6180 (38.59)	341 (37.85)	3932 (28.17)	1861 (32.61)
Transient ischemic attack	94 (23.04)	7596 (17.85)	3084 (19.26)	150 (16.65)	1665 (11.93)	819 (14.35)
Alcoholism	18 (4.41)	1324 (3.11)	663 (4.14)	34 (3.77)	397 (2.84)	221 (3.87)
Peripheral artery disease	86 (21.08)	7461 (17.53)	3296 (20.58)	173 (19.20)	2173 (15.57)	1109 (19.43)
Coronary artery disease	213 (52.21)	20 951 (49.23)	9018 (56.32)	483 (53.61)	6081 (43.57)	2845 (49.85)
Stroke/SE	94 (23.04)	6520 (15.32)	3007 (18.78)	157 (17.43)	1436 (10.29)	856 (15.00)
All‐cause hospitalization, n (%)	264 (64.71)	21 908 (51.48)	11 874 (74.15)	591 (65.59)	5853 (41.94)	3676 (64.41)
Bleeding event between index date and switch/pseudo‐switch date, n (%)	62 (15.20)	4158 (9.77)	…	252 (27.97)	1163 (8.33)	…
Days from DOAC initiation date to index date						
Mean±SD	162.00±252.96	280.03±329.29	…	199.71±275.17	295.88±360.08	…
Median (IQR)	82.5 (41.5–157.5)	159 (73–358)	…	97 (55–230)	162 (75–372)	…
Days from index bleeding event to index date						
Mean±SD)	35.84±26.32	35.83±26.29	…	33.83±26.26	33.83±26.25	…
Median (IQR)	29.5 (13–59)	30 (13–59)	…	28 (10–54)	28 (10–54)	…
Days from DOAC initiation date to index bleeding event						
Mean±SD	126.16±249.31	244.20±328.19	181.14±290.11	165.88±275.28	262.05±359.21	168.26±295.59
Median (IQR)	38 (11–115.5)	120 (32–321)	67 (19–212)	55 (18–193)	126 (36–337)	57 (17–182)
Days of follow‐up						
Mean±SD	304.13±379.34	372.86±405.76	…	382.65±477.60	442.25±499.86	…
Median (IQR)	167 (59–343)	225 (105–496)	…	207 (82–467)	256 (115–580)	…
Index DOAC dose, n (%)						
Standard dose†	341 (83.58)	34 383 (80.79)	11 940 (74.56)	676 (75.03)	10 684 (76.55)	3791 (66.43)
Low dose‡	67 (16.42)	8177 (19.21)	4073 (25.44)	225 (24.97)	3273 (23.45)	1916 (33.57)
DOAC initiation index year, n (%)						
2013	<5*	163 (0.38)	54 (0.34)	27 (3.00)	1066 (7.64)	482 (8.45)
2014	10 (2.45)	705 (1.66)	203 (1.27)	42 (4.66)	1157 (8.29)	526 (9.22)
2015	13 (3.19)	1383 (3.25)	415 (2.59)	33 (3.66)	1084 (7.77)	403 (7.06)
2016	15 (3.68)	2146 (5.04)	713 (4.45)	66 (7.33)	1168 (8.37)	458 (8.03)
2017	33 (8.09)	3157 (7.42)	1018 (6.36)	85 (9.43)	15811 (1.33)	581 (10.18)
2018	40 (9.80)	4711 (11.07)	1676 (10.47)	117 (12.99)	1734 (12.42)	691 (12.11)
2019	75 (18.38)	5547 (13.03)	1949 (12.17)	119 (13.21)	1500 (10.75)	572 (10.02)
2020	43 (10.54)	5683 (13.35)	2154 (13.45)	100 (11.10)	1206 (8.64)	480 (8.41)
2021	66 (16.18)	7813 (18.36)	2873 (17.94)	133 (14.76)	1674 (11.99)	659 (11.55)
2022	56 (13.73)	6652 (15.63)	2713 (16.94)	106 (11.76)	1097 (7.86)	522 (9.15)
2023	53 (12.99)	4600 (10.81)	2245(14.02)	73 (8.10)	690 (4.94)	333 (5.83)

CCI indicates Charlson Comorbidity Index; DOAC, direct oral anticoagulant; IQR, interquartile range; and SE, systemic embolism.

* To preserve patient anonymity, values of n <5 are given as “<5” (and the corresponding percentage is not shown).

^†^ 5 mg for apixaban; 20 mg for rivaroxaban.

^‡^ 2.5 mg for apixaban; 10 or 15 mg for rivaroxaban.

Across apixaban initiator cohorts, >70% of patients had a CHA_2_DS_2_‐VASc score ≥4, indicating a high baseline risk of stroke. Compared with persistent apixaban users, apixaban‐to‐rivaroxaban switchers had a higher mean CCI score (4.26 versus 3.83), a higher mean CHA_2_DS_2_‐VASc score (4.82 versus 4.57) and greater proportion of patients with a CHA_2_DS_2_‐VASc score ≥4 (78.19% versus 73.05%), and a higher mean HAS‐BLED score (4.09 versus 3.90) and greater proportion of patients with a HAS‐BLED score ≥3 (93.87% versus 91.75%). They also had higher frequencies of congestive heart failure (45.10% versus 40.10%), liver disease (13.24% versus 9.58%), transient ischemic attack (23.04% versus 17.85%), and stroke/SE (23.04% versus 15.32%); a greater frequency of all‐cause hospitalization (64.71% versus 51.48%); and a shorter mean interval from DOAC initiation to the index bleeding event (126.16 versus 244.20 days).

Compared with persistent apixaban users, impersistent users had a similar mean age (76.91 versus 75.93 years), a similar proportion of female patients (47.34% versus 49.72%), a higher mean CCI score (4.87 versus 3.83), slightly higher mean CHA_2_DS_2_‐VASc (4.88 versus 4.57) and HAS‐BLED (4.18 versus 3.90) scores, a higher frequency of previous stroke/SE (18.78% versus 15.32%), and higher frequences of multiple other comorbidities.

### Rivaroxaban Initiators

For >85% of rivaroxaban switchers, persistent users, and impersistent users, the index bleeding event was clinically relevant nonmajor bleeding (Table 1). Most index bleeding events (>50% for rivaroxaban switchers and impersistent users, 71% for persistent rivaroxaban users) were of types other than ICH and gastrointestinal bleeding. Compared with persistent rivaroxaban users, greater proportions of impersistent users had ICH or multiple types of bleeding as the index bleeding event.

Across rivaroxaban initiator cohorts, >60% of patients had a CHA_2_DS_2_‐VASc score ≥4. Compared with persistent rivaroxaban users, rivaroxaban‐to‐apixaban switchers had a higher mean CCI score (4.11 versus 3.01); a higher mean CHA_2_DS_2_‐VASc score (4.64 versus 4.06) and greater proportion of patients with a CHA_2_DS_2_‐VASc score ≥ 4 (75.14% versus 61.38%), and a higher mean HAS‐BLED score (3.99 versus 3.59) and greater proportion of patients with a HAS‐BLED score ≥3 (93.23% versus 86.42%). They also had higher frequencies of multiple comorbidities, including stroke/SE (17.43% versus 10.29%), a greater frequency of all‐cause hospitalization (65.59% versus 41.94%), and a shorter mean interval from DOAC initiation to the index bleeding event (165.88 versus 262.05 days).

Compared with persistent rivaroxaban users, impersistent users were slightly older (mean age 75.04 versus 73.88 years) but had a similar proportion of female patients (43.72% versus 44.62%), had a higher mean CCI score (3.89 versus 3.01) and slightly higher mean CHA_2_DS_2_‐VASc (4.41 versus 4.06) and HAS‐BLED (3.86 versus 3.59) scores, and had higher frequencies of previous stroke/SE (15.00% versus 10.29%) and multiple other comorbidities.

### Patient Characteristics After PSM

To facilitate comparison of DOAC switchers with persistent users, the 408 apixaban‐to‐rivaroxaban switchers were matched with 2040 persistent apixaban users, and the 901 rivaroxaban‐to‐apixaban switchers with 4505 persistent rivaroxaban users (Table 2). Following PSM, the proportion of patients who were hospitalized was higher for rivaroxaban‐to‐apixaban switchers than for persistent rivaroxaban users (65.59% versus 53.98%; SMD, 23.84). Moreover, the proportion of patients who had a bleeding event between the index bleeding event and the switch/pseudo‐switch date was higher for apixaban‐to‐rivaroxaban switchers compared with persistent apixaban users (15.20% versus 11.42%; SMD, 11.13) and for rivaroxaban‐to‐apixaban switchers compared with persistent rivaroxaban users (27.97% versus 11.37%; SMD, 42.71). Otherwise, switchers and persistent users were generally well matched across cohorts after PSM (SMD ≤10) (Figure S1). Across PSM‐matched cohorts, the index DOAC dose for most patients was the standard dose. Reasons for censoring apixaban and rivaroxaban initiators in the PSM‐matched cohorts are shown in Tables S5 and S6, respectively.

**Table 2:** Post‐PSM Baseline Characteristics of Switchers and Persistent Users

	Apixaban initiators					Rivaroxaban initiators				
	Apixaban‐to‐rivaroxaban switchers (N=408)		Persistent apixaban users (N=2040)		SMD*	Rivaroxaban‐to‐apixaban switchers (N=901)		Persistent rivaroxaban users (N=4505)		SMD*
Age, y†										
Mean (SD)	75.43	8.16	75.60	9.20	1.69	74.92	9.30	74.23	9.50	7.44
Median (IQR)	76	70, 81.5	77	70, 83		76	70, 82	75	69, 82	
Age category, y, n (%)										
18 to 54	5	1.23%	48	2.35%	11.19	31	3.44%	154	3.42%	11.33
55 to 64	31	7.60%	149	7.30%		69	7.66%	435	9.66%	
65 to 74	125	30.64%	618	30.29%		271	30.08%	1489	33.05%	
75 to 79	94	23.04%	428	20.98%		198	21.98%	863	19.16%	
≥80	153	37.50%	797	39.07%		332	36.85%	1564	34.72%	
Sex, n (%)†										
Female	213	52.21%	1027	50.34%	3.73	419	46.50%	1979	43.93%	6.03
Male	195	47.79%	1013	49.66%		482	53.50%	2524	56.03%	
Unknown	0	0.00%	0	0.00%		0	0.00%	<5‡	‐	
Site/type of index bleeding event, n (%)										
Gastrointestinal	90	22.06%	455	22.30%	4.10	197	21.86%	910	20.20%	4.93
Intracranial hemorrhage	28	6.86%	122	5.98%		29	3.22%	153	3.40%	
Other	259	63.48%	1343	65.83%		528	58.60%	3139	69.68%	
Multiple	31	7.60%	120	5.88%		147	16.32%	303	6.73%	
Severity of index bleeding event, n (%)										
Major bleeding	26	6.37%	92	4.51%		119	13.21%	193	4.28%	
Clinically relevant non‐major bleeding	382	93.63%	1948	95.49%		782	86.79%	4312	95.72%	
CCI score†										
Mean (SD)	4.26	2.87	4.33	3.04	2.34	4.11	2.93	4.11	2.89	0.04
Median (IQR)	4	2, 6	4	2, 6		4	2, 6	4	2, 6	
CHA_2_DS_2_‐VASc score										
Mean (SD)	4.82	1.72	4.83	1.69	0.78	4.64	1.70	4.53	1.75	6.10
Median (IQR)	5	4, 6	5	4, 6		5	4, 6	4	3, 6	
CHA_2_DS_2_‐VASc score, n (%)										
0	<5‡	‐	6	0.29%	0.00	<5‡	‐	16	0.36%	12.12
1	8	1.96%	39	1.91%		25	2.77%	131	2.91%	
2	26	6.37%	124	6.08%		68	7.55%	397	8.81%	
3	53	12.99%	266	13.04%		127	14.10%	793	17.60%	
≥4	319	78.19%	1605	78.68%		677	75.14%	3168	70.32%	
HAS‐BLED score										
Mean (SD)	4.09	1.14	4.04	1.14	4.61	3.99	1.10	3.97	1.12	2.05
Median (IQR)	4	3, 5	4	3, 5		4	3, 5	4	3, 5	
HAS‐BLED score, n (%)										
0	0	0.00%	0	0.00%	4.39	0	0.00%	0	0.00%	7.85
1	<5‡	‐	15	0.74%		9	1.00%	29	0.64%	
2	22	5.39%	123	6.03%		52	5.77%	348	7.72%	
≥3	383	93.87%	1902	93.24%		840	93.23%	4128	91.63%	
Baseline comorbidities, n (%)†										
History of any bleeding	408	100.00%	2040	100.00%	0.00	901	100.00%	4505	100.00%	0.00
Congestive heart failure	184	45.10%	981	48.09%	6.00	394	43.73%	2001	44.42%	1.39
Diabetes	169	41.42%	834	40.88%	1.10	352	39.07%	1772	39.33%	0.55
Hypertension	377	92.40%	1887	92.50%	0.37	844	93.67%	4184	92.87%	3.19
Renal disease	177	43.38%	845	41.42%	3.97	417	46.28%	2088	46.35%	0.13
Liver disease	54	13.24%	271	13.28%	0.14	112	12.43%	528	11.72%	2.18
Myocardial infarction	73	17.89%	379	18.58%	1.78	162	17.98%	797	17.69%	0.75
Dyspepsia or stomach discomfort	109	26.72%	573	28.09%	3.08	188	20.87%	913	20.27%	1.48
Peripheral vascular disease	158	38.73%	815	39.95%	2.51	341	37.85%	1679	37.27%	1.19
Transient ischemic attack	94	23.04%	481	23.58%	1.28	150	16.65%	741	16.45%	0.54
Alcoholism	18	4.41%	93	4.56%	0.71	34	3.77%	173	3.84%	0.35
Peripheral arterial disease	86	21.08%	436	21.37%	0.72	173	19.20%	868	19.27%	0.17
Coronary artery disease	213	52.21%	1100	53.92%	3.44	483	53.61%	2420	53.72%	0.22
Stroke/systemic embolism	94	23.04%	470	23.04%	0.00	157	17.43%	801	17.78%	0.93
All‐cause hospitalization, n (%)	264	64.71%	1294	63.43%	2.66	591	65.59%	2432	53.98%	23.84
Bleeding event between index date and switch/pseudo‐switch date, n (%)	62	15.20%	233	11.42%	11.13	252	27.97%	512	11.37%	42.71
Days from DOAC initiation date to index date										
Mean (SD)	162.00	252.96	168.23	239.25	2.53	199.71	275.17	198.70	229.53	0.40
Median (IQR)	82.5	41.5, 157.5	95.5	52, 179		97	55, 230	115	61, 248	
Days from index bleeding event to index date†										
Mean (SD)	35.84	26.32	36.23	26.61	1.48	33.83	26.26	33.54	26.08	1.12
Median (IQR)	29.5	13, 59	30	13, 59		28	10, 54	28	10, 54	
Days from DOAC initiation date to index bleeding event†										
Mean (SD)	126.16	249.31	132.00	237.25	2.40	165.88	275.28	165.16	227.99	0.28
Median (IQR)	38	11, 115.5	55	16, 136		55	18, 193	79	24, 211	
Days of follow‐up										
Mean (SD)	304.13	379.34	345.14	396.23	10.57	382.65	477.60	394.29	462.36	2.48
Mean (IQR)	167	59, 343	203	95.5, 423.5		207	82, 467	222	102, 499	
Index DOAC dose, n (%)										
Standard dose§	341	83.58%	1689	82.79%	2.10	676	75.03%	3269	72.56%	4.63
Low dose||	67	16.42%	351	17.21%		225	24.97%	1236	27.44%	
DOAC Initiation index year, n (%)										
2013	<5‡	‐	7	0.34%	24.11	27	3.00%	293	6.50%	32.36
2014	10	2.45%	24	1.18%		42	4.66%	346	7.68%	
2015	13	3.19%	57	2.79%		33	3.66%	280	6.22%	
2016	15	3.68%	90	4.41%		66	7.33%	353	7.84%	
2017	33	8.09%	152	7.45%		85	9.43%	477	10.59%	
2018	40	9.80%	244	11.96%		117	12.99%	592	13.14%	
2019	75	18.38%	260	12.75%		119	13.21%	521	11.56%	
2020	43	10.54%	240	11.76%		100	11.10%	408	9.06%	
2021	66	16.18%	333	16.32%		133	14.76%	568	12.61%	
2022	56	13.73%	341	16.72%		106	11.76%	398	8.83%	
2023	53	12.99%	292	14.31%		73	8.10%	269	5.97%	

CCI indicates Charlson Comorbidity Index; DOAC, direct oral anticoagulant; IQR, interquartile range; PSM, propensity score matching; and SMD, standardized mean difference (a value >10 is considered significant).

* Calculated by comparing switchers with persistent users.

^†^ Variables used for propensity score matching.

^‡^ To preserve patient anonymity, values of n <5 are given as “<5” (and the corresponding percentage is not shown).

^§^ 5 mg for apixaban; 20 mg for rivaroxaban.

^‖^ 2.5 mg for apixaban; 10 or 15 mg for rivaroxaban.

### Comparative Analysis of Outcomes After PSM

**Figure 3:**
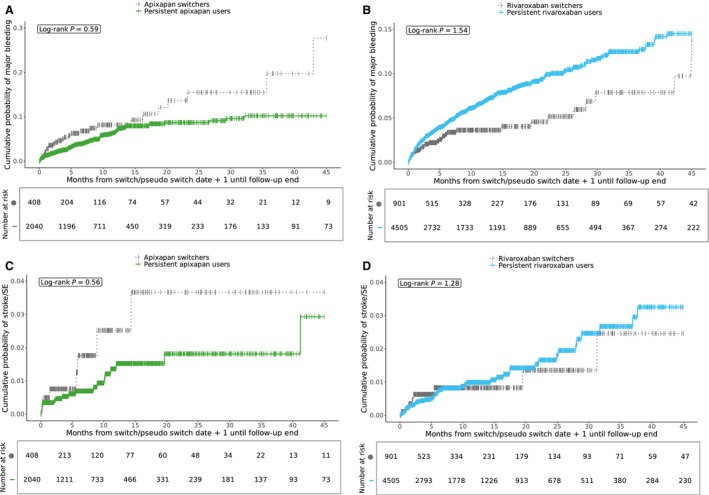
Cumulative incidence curves for major bleeding and stroke/SE. Cumulative incidence curves for apixaban initiators (**A, C**) and rivaroxaban initiators (**B, D**). SE indicates systemic embolism.

Cumulative incidence curves for time to major bleeding and time to stroke/SE among apixaban and rivaroxaban initiators are shown in Figure 3. For all models, Schoenfeld’s *P* value was >0.05, indicating that the proportional hazards assumption was met (Figure S2).

### Apixaban Initiators

**Figure 4:**
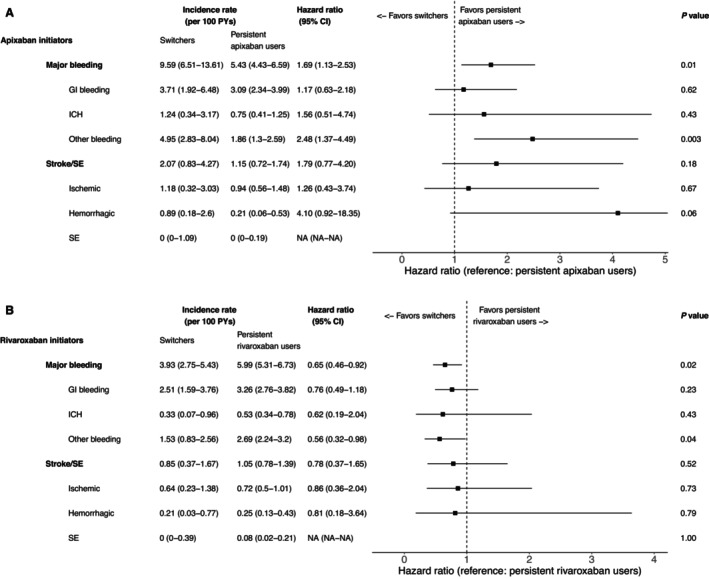
Comparison of risks of major bleeding and stroke/SE. Comparisons between apixaban‐to‐rivaroxaban switchers and persistent apixaban users (**A**) and between rivaroxaban‐to‐apixaban switchers and persistent rivaroxaban users (**B**). Incidence rates are per 100 patient‐years. Persistent users were the reference group in hazard ratio calculations. *Hazard ratio, 95% CI, and *P* value not calculable because of 1 or both cohorts having 0 events. For apixaban initiators, the 95% CI for hemorrhagic stroke is truncated in the interests of providing a clear visualization of the data. GI indicates gastrointestinal; ICH, intracranial hemorrhage; NA, not available; and SE, systemic embolism.

The incidence rate for major bleeding was 9.59 per 100 patient‐years for apixaban‐to‐rivaroxaban switchers and 5.43 per 100 patient‐years for persistent apixaban users (Figure 4A). Compared with persisting with apixaban, switching to rivaroxaban was associated with significantly higher risks of major bleeding (HR, 1.690 [95% CI, 1.130–2.527]) and other bleeding (ie, bleeding other than gastrointestinal bleeding and ICH: HR, 2.481 [95% CI, 1.372–4.485]); risks of gastrointestinal bleeding (HR, 1.172 [95% CI, 0.629–2.182]) and ICH (HR, 1.559 [95% CI, 0.513–4.740]) were similar among apixaban‐to‐rivaroxaban switchers and persistent apixaban users. For stroke/SE, the incidence rate was 2.07 per 100 patient‐years for apixaban‐to‐rivaroxaban switchers and 1.15 per 100 patient‐years for persistent apixaban users. The risk of stroke/SE was similar for apixaban‐to‐rivaroxaban switchers and persistent apixaban users (HR, 1.793 [95% CI, 0.765–4.202]), as were the risks of ischemic stroke (HR, 1.265 [95% CI, 0.428–3.740]) and hemorrhagic stroke (HR, 4.101 [95% CI, 0.916–18.351]; Figure 4A).

### Rivaroxaban Initiators

Among rivaroxaban initiators, the incidence rate for major bleeding was 3.93 per 100 patient‐years for rivaroxaban‐to‐apixaban switchers and 5.99 per 100 patient‐years for persistent rivaroxaban users (Figure 4B). Compared with persisting with rivaroxaban, switching to apixaban was associated with significantly lower risks of major bleeding (HR, 0.651 [95% CI, 0.460–0.920]) and other bleeding (HR, 0.564 [95% CI, 0.325–0.980]); risks of gastrointestinal bleeding (HR, 0.763 [95% CI, 0.492–1.182]) and ICH (HR, 0.616 [95% CI, 0.186–2.040]) were similar among rivaroxaban‐to apixaban switchers and persistent rivaroxaban users. The incidence rate for stroke/SE was 0.85 per 100 patient‐years for rivaroxaban‐to‐apixaban switchers and 1.05 per 100 patient‐years for persistent rivaroxaban users. The risk of stroke/SE was similar for rivaroxaban‐to‐apixaban switchers and persistent rivaroxaban users (HR, 0.783 [95% CI, 0.371–1.652]), as were the risks of ischemic stroke (HR, 0.857 [95% CI, 0.360–2.042]) and hemorrhagic stroke (HR, 0.814 [95% CI, 0.182–3.641]; Figure 4B).

### Sensitivity Analyses

When Cox models were adjusted for variables that remained imbalanced after PSM (all‐cause hospitalization [rivaroxaban initiators] and bleeding between the index date and switch/pseudo‐switch date [apixaban and rivaroxaban initiators]), the effectiveness and safety results were similar to those from the main model. Compared with persisting with apixaban, switching to rivaroxaban was associated with an increased risk of major bleeding (HR, 1.525 [95% CI, 1.016–2.287]) and a similar risk of stroke/SE (HR, 1.685 [95% CI, 0.715–3.969]; Table 3). Compared with persisting with rivaroxaban, switching to apixaban was associated with a reduced risk of major bleeding (HR, 0.511 [95% CI, 0.359–0.727]) and a similar risk of stroke/SE (HR, 0.610 [95% CI, 0.285–1.305]; Table 3).

**Table 3:** Risks of Major Bleeding and Stroke/SE in Apixaban‐to‐Rivaroxaban Switchers and Rivaroxaban‐to‐Apixaban Switchers Calculated With Adjustment for Variables That Were Imbalanced After PSM*

	Hazard ratio	95% CI	*P* value
Apixaban‐to‐rivaroxaban switchers vs persistent apixaban users			
Major bleeding	1.525	1.016–2.287	0.0414
Stroke/SE	1.685	0.715–3.969	0.2328
Rivaroxaban‐to‐apixaban switchers vs persistent rivaroxaban users			
Major bleeding	0.511	0.359–0.727	0.0002
Stroke/SE	0.610	0.285–1.305	0.2026

PSM indicates propensity score matching; and SE, systemic embolism.

* All‐cause hospitalization (rivaroxaban initiators) and bleeding between the index date and switch/pseudo‐switch date (apixaban and rivaroxaban initiators).

In a second sensitivity analysis in which death was included as a competing risk, the results were also consistent with those from the main models. Compared with persisting with apixaban, switching to rivaroxaban was associated with an increased risk of major bleeding (HR, 1.683 [95% CI, 1.130–2.507]) and a similar risk of stroke/SE (HR, 1.829 [95% CI, 0.784–4.268]; Table 4). Compared with persisting with rivaroxaban, switching to apixaban was associated with a reduced risk of major bleeding (HR, 0.653 [95% CI, 0.465–0.917]) and a similar risk of stroke/SE (HR, 0.783 [95% CI, 0.367–1.668]; Table 4).

**Table 4:** Risks of Major Bleeding and Stroke/SE in Apixaban‐to‐Rivaroxaban Switchers and Rivaroxaban‐to‐Apixaban Switchers With Death Included as a Competing Risk

	Hazard ratio	95% CI	*P* value
Apixaban‐to‐rivaroxaban switchers vs persistent apixaban users			
Major bleeding	1.683	1.130–2.507	0.0104
Stroke/SE	1.829	0.784–4.268	0.1627
Rivaroxaban‐to‐apixaban switchers vs persistent rivaroxaban users			
Major bleeding	0.653	0.465–0.917	0.0139
Stroke/SE	0.783	0.367–1.668	0.5255

SE indicates systemic embolism.

E‐values were calculated to assess unmeasured confounding. For apixaban initiators, the E‐value (lower bound of the 95% CI for the HR) was 2.230 (1.399) for major bleeding and 2.359 (1.000) for stroke/SE (Table S7). For rivaroxaban initiators, the E‐value (upper bound of the 95% CI for the HR) was 2.029 (1.310) for major bleeding and 1.652 (1.000) for stroke/SE.

## DISCUSSION

In this study of postbleeding anticoagulant treatment in patients with NVAF who received DOACs to reduce their risk of stroke, switching from apixaban to rivaroxaban was associated with a higher risk of major bleeding compared with persisting with apixaban. By contrast, switching from rivaroxaban to apixaban was associated with a lower risk of major bleeding compared with persisting with rivaroxaban. For both apixaban and rivaroxaban initiators, the risk of stroke/SE was similar for patients who switched their DOAC as for those who persisted with the same DOAC. Current US treatment guidelines lack specific recommendations for selecting a DOAC after a bleeding event^8^ due to the need to consider various factors, such as bleeding site and severity and patient risk, as well as insufficient evidence on risks and benefits of switching the DOAC after bleeding. Thus, findings from this study may provide useful insights for physicians on DOAC selection after bleeding.

The current study findings also provide insights into physician prescribing habits. The higher disease burden among apixaban initiators compared with rivaroxaban initiators indicates that physicians may be more likely to prescribe apixaban to higher‐risk patients. Moreover, the low proportion of apixaban switchers versus rivaroxaban switchers (1.8% versus 6.4%) among the respective initiator cohorts may reflect physician hesitance to switch away from apixaban following a bleeding event. These apparent physician preferences may be based on the body of evidence supporting the safety and effectiveness of apixaban in patients with AF, including high‐risk patient subgroups.^18^, ^19^, ^20^, ^30^, ^31^, ^32^, ^33^, ^34^

The observation that about a quarter of apixaban and rivaroxaban initiators were impersistent users highlights a potential care gap. Given the large proportions of included patients who had a high stroke risk on the basis of CHA_2_DS_2_‐VASc, the fact that so many patients did not resume anticoagulant therapy within 90 days after their index bleeding event warrants further investigation. Potential contributing factors could include concerns about recurrent bleeding; the site, severity, and mechanism of bleeding (ie, traumatic or spontaneous); the source of bleeding and whether it was definitively identified and controlled; patient characteristics; and comorbid conditions.^35^ A lack of clear guidelines and suboptimal transition of care between specialist care providers and primary care physicians may also have played a role. Additionally, it should be noted that some patients may have undergone left atrial appendage closure following the bleeding event; however, further research is needed to clarify the role of this procedure in the context of postbleeding treatment nonpersistence. Current US treatment guidelines recommend that resumption of OAC treatment can be considered in patients with AF who experience major gastrointestinal bleeding or ICH while receiving anticoagulation.^8^ They further indicate that OAC resumption is clinically beneficial in most cases,^35^ a conclusion supported by a meta‐analysis of studies with data for OAC restarters and nonrestarters.^36^ An American College of Cardiology consensus decision pathway advocates prompt resumption of OAC treatment in patients with a high risk of thrombosis.^35^ For many patients, permanently discontinuing anticoagulant treatment may not be the optimal response to a bleeding event. This may be especially true for patients whose index bleeding event is clinically relevant non‐major bleeding, as was the case for most patients in the present study (in which clinically relevant non‐major bleeding was classified as not having a primary inpatient diagnosis). Bleeding risk should not be the only factor in deciding whether to continue treating patients with anticoagulants. Rather, physicians should work with patients and the multidisciplinary care team to weigh the net clinical benefit of OAC resumption on a case‐by‐case basis and to ensure that patients with a high bleeding risk are closely monitored if therapy is restarted.

The present findings are aligned with and extend the findings of a previous retrospective analysis conducted using the same data source, but where included patients were not required to have a claim for bleeding.^22^ This earlier study found that apixaban‐to‐rivaroxaban switching was associated with a higher risk of major bleeding and rivaroxaban‐to‐apixaban switching with lower risk of major bleeding, suggesting that safety outcomes depend on which DOAC patients are switched from and to. However, in the earlier study, reasons for DOAC switching were not evaluated.

In the present study, the elevated risk of major bleeding in patients who switched from apixaban to rivaroxaban, and reduction of major bleeding risk in patients who switched from rivaroxaban to apixaban, were primarily driven by bleeding events other than gastrointestinal bleeding and ICH. This observation likely reflects the predominance of other bleeding among index bleeding events in apixaban and rivaroxaban initiator cohorts. This is not surprising, as our study was designed to reflect real‐world clinical practice, where most bleeding events among patients with NVAF on DOACs are nonfatal and extracranial.^37^ However, these bleeding events are relevant enough to prompt treatment reassessment. The inclusion of a broader spectrum of bleeding events allows for a more comprehensive understanding of physician decision‐making and patient outcomes by comparing patients who persisted with their index DOAC with those who switched to an alternative DOAC.

### Strengths and Limitations

Strengths of the present study include the rigorous statistical design, with PSM minimizing bias by ensuring that patient characteristics and baseline event rates were comparable between switcher and persistent user cohorts. Nonetheless, there may have been unmeasured confounding, which we assessed by calculating E‐values. Despite PSM, however, proportions of patients who had a bleeding event between the index bleeding event and the switch/pseudo‐switch date remained imbalanced between switcher and persistent user cohorts. However, findings from a sensitivity analysis that adjusted for post‐PSM imbalances were consistent with the primary analysis.

As with other claims‐based studies, there are some important limitations to consider. Although we were able to track continuous treatment exposure through prescription records in Optum CDM, certain covariates such as comorbidities, health care use, and disease progression can change over time. If these changes are not captured, they may introduce confounding. We used PSM to account for baseline differences between cohorts. However, this approach does not account for changes in covariates during follow‐up, which may still influence outcomes. Additionally, the use of diagnostic codes may have led to misclassification of some conditions or outcomes. While we applied rigorous methods to reduce bias, these limitations should be kept in mind when interpreting the results.

The unmatched and post‐PSM samples were smaller than in other real‐world OAC studies^18^, ^19^, ^22^ because of the scope of the present analyses being limited to patients with at least 1 prior bleeding event. Notably, there were too few patients for a meaningful analysis of postbleeding DOAC selection and treatment outcomes in dabigatran and edoxaban initiators. Nonetheless, power calculations indicated that the study was adequately powered to detect significant differences on the primary outcome (major bleeding). However, the analyses of gastrointestinal bleeding and other individual components of the composite major bleeding and stroke/SE outcomes were likely underpowered.

In limiting the analyses to patients who switched DOAC following a bleeding event, it was assumed that the bleeding event was the cause of DOAC switching. However, this may not have been the only clinical reason for a patient to switch to a new DOAC, given that bleeding risks are multifactorial.^38^ In some cases, ≥1 other unidentified clinical or nonclinical factors may have been the cause of switching.

Following PSM, there was still a higher proportion of hospitalized patients among rivaroxaban‐to‐apixaban switchers compared with persistent rivaroxaban users. However, because this imbalance favored persistent rivaroxaban users, any effect on the HRs comparing rivaroxaban switchers versus persistent users was likely conservative.

While a change in AF practice during the study period may have had an impact on the results, the distribution around the DOAC initiation index year was well balanced between the switcher and persistent user cohorts. Additionally, most patients had an index year within the past 5 years, and thus, the impact of any change in AF practice would have been low.

As with other observational studies, while statistical associations between exposures and outcomes could be established, causality could not be inferred. Also, because DOAC use was based on pharmacy fills, it was not possible to determine whether patients took their DOAC(s) as prescribed. Finally, patients were selected using specific inclusion and exclusion criteria, which may limit generalizability of the findings to a broader population. Optum CDM includes data only for patients with commercial employer‐sponsored insurance or Medicare Advantage. Generalizability of the present findings to patients with other types of insurance or no insurance is unclear. Generalizability to patients outside of the United States is likewise uncertain, especially given the recognized ethnic differences in AF‐related complications, such as stroke and bleeding.^39^, ^40^ Indeed, Asian patients may demonstrate a greater propensity to bleeding with antithrombotic drugs, the so‐called "East Asian paradox."^41^

## CONCLUSIONS

In conclusion, after a bleeding event, the risk of major bleeding was higher when switching from apixaban to rivaroxaban than when persisting with apixaban. Conversely, the risk of major bleeding was lower when switching from rivaroxaban to apixaban than when persisting with rivaroxaban. Risks of stroke/SE were similar for patients who switched their DOAC and those who persisted with their index DOAC. The reasons for high proportions of patients not being anticoagulated within 90 days of a bleeding event, despite a high risk of stroke, warrant further investigation.

## Sources of Funding

This study was sponsored by the Pfizer and Bristol Myers Squibb Alliance. The funders had no role in study design, data collection and analysis, decision to publish, or preparation of the manuscript.

## Disclosures

Dr Lip received nonpersonal research support from the Pfizer and Bristol Myers Squibb Alliance, the study sponsors, in connection with the conduct of this study. Dr Lip reported serving as a consultant and speaker for BMS/Pfizer, Boehringer Ingelheim, Daiichi‐Sankyo, and Anthos outside the submitted work (no personal fees). Dr Lip is a National Institute for Health and Care Research Senior Investigator Emeritus and co‐principal investigator of the AFFIRMO project on multimorbidity in AF, which has received funding from the European Union’s Horizon 2020 research and innovation program under grant agreement no. 899871. Dr Deitelzweig received research support from the Pfizer and Bristol Myers Squibb Alliance, the study sponsors, in connection with the conduct of this study. Dr Deitelzweig is a consultant for Bayer/Janssen, Bristol Myers Squibb Company/Pfizer Inc, Daiichi‐Sankyo, and Gilead and has been on the speakers’ bureau for Janssen, Bristol Myers Squibb Company/Pfizer Inc. Drs Cheng, Jiang, Gao, and Dubey are employees and shareholders of Bristol Myers Squibb, a study sponsor. R. Subash and Dr Vodicka are employees and shareholders of Pfizer, a study sponsor. Dr Sharma was an employee of Pfizer during the conduct of this study. Anakha Vettumthara Unnikrishnan and Kishorekumar Mathivanan are employees of Mu Sigma Inc., which received payment from Bristol Myers Squibb in connection with the conduct of this study.

## Supporting information

Data S1
Tables S1–S7
Figures S1–S2
