# Supplementary material for: Clinical Impact of Switching From or Persisting With Rivaroxaban or Apixaban After a Bleeding Event: A Real‐World Study in Patients With Nonvalvular Atrial Fibrillation
Source: J Am Heart Assoc. 2026 Jan 19;15(2):e044113. doi: 10.1161/JAHA.125.044113 (PMC12919467; doi:10.1161/JAHA.125.044113)
Supplement: Supplementary file 1 — Data S1 Tables S1–S7 Figures S1–S2 [file JAH3-15-e044113-s001.pdf]

Supporting Information

Data S1.

As it was anticipated that Cox regression models would be fitted to the data, the following formula was used for the power calculations:

$$1 - \beta = \Phi^{-1} \left(\sqrt{(NP_A\, P_B\, P_E\,)} \times \ln(HR)\right) - z_{(1-\alpha/2)}$$

...where α is the probability of a type I error, β is the probability of a type II error, N is the total sample size, P_E is the overall event rate, P_A and P_B are proportions in the two groups, HR is the expected hazard ratio, z is the score from the standard normal distribution, and Φ is the standard normal distribution. The formula was used to derive the HRs that could be detected with an α of 0.05 and a power (i.e., value for $1 - \beta$) of 80%. A 1-year observation time for each group was assumed. For apixaban initiators, based on a preliminary unadjusted major bleeding rate of 9.56% in the switcher cohort and an HR of 1.80 observed in a previous study [22], it was determined that 394 apixaban-to-rivaroxaban switchers and 1,970 matched persistent apixaban users would be needed to ensure a sufficient sample size to detect significant differences. For rivaroxaban initiators, based on a preliminary unadjusted major bleeding rate of 3.54% in the switcher cohort and an HR of 0.49 observed in a previous study [22], it was determined that 436 rivaroxaban-to apixaban switchers and 2,180 matched persistent rivaroxaban users would be needed to ensure a sufficient sample size to detect significant differences.

Table S1. National Drug Codes used to identify oral anticoagulants

Generic name	National Drug Codes
Apixaban	00003089321, 00003089331, 00003089391, 00003089421, 00003089431, 00003089470, 00003089491, 00003376432, 00003376442, 00003376474, 50090143600, 50090143700, 54569651300, 54569651400, 55154061200, 55154061208, 55154061300, 55154061308
Dabigatran etexilate mesylate	00597010754, 00597010760, 00597010854, 00597010860, 00597013554, 00597013560, 00597014954, 00597014960, 00597035509, 00597035556, 00597036042, 00597036055, 00597036082, 21695089960, 31722062160, 31722062260, 50090448000, 54569627600, 67877047460, 67877047560
Edoxaban tosylate	65597020130, 65597020205, 65597020210, 65597020230, 65597020290, 65597020305, 65597020310, 65597020330, 65597020390
Rivaroxaban	42254037601, 50090363900, 50090446800, 50090446900, 50458057501, 50458057701, 50458057710, 50458057714, 50458057718, 50458057760, 50458057801, 50458057810, 50458057830, 50458057890, 50458057901, 50458057910, 50458057930, 50458057989, 50458057990, 50458058001, 50458058010, 50458058030, 50458058090, 50458058451, 55154142200, 55154142308, 55154142400, 55154142408
Warfarin sodium	00034542080, 00056016801, 00056016870, 00056016875, 00056016890, 00056016901, 00056016970, 00056016975, 00056016990, 00056017001, 00056017030, 00056017070, 00056017075, 00056017090, 00056017170, 00056017175, 00056017190, 00056017201, 00056017230, 00056017270, 00056017275, 00056017290, 00056017301, 00056017370, 00056017375, 00056017401, 00056017470, 00056017475, 00056017601, 00056017630, 00056017670, 00056017675, 00056017690, 00056018801, 00056018870, 00056018875, 00056018890, 00056018901, 00056018970, 00056018975, 00056018990, 00056033006, 00074662603, 00074662607, 00074663803, 00074663807, 00074720201, 00074720205, 00074721001, 00074721005, 00074721009, 00074721801, 00074721805, 00093014301, 00093014401, 00093014501, 00093014510, 00093171201, 00093171210, 00093171301, 00093171310, 00093171401, 00093171410, 00093171501, 00093171601, 00093171610, 00093171801, 00093171901, 00093172001, 00093172101, 00093172110, 00093172301, 00150256060, 00150256160, 00150256260, 00150256360, 00150256460, 00157028301, 00157028401, 00157028501, 00157028510, 00157028601, 00157028701, 00182152001, 00182152101, 00182152201, 00182152210, 00182152301, 00182152401, 00182267101, 00182267110, 00182267189, 00182267201, 00182267210, 00182267289, 00182267301, 00182267310, 00182267389, 00182267401, 00182267489, 00182267501, 00182267589, 00182267601, 00182267610, 00182267689, 00182267701, 00182267789, 00182267801, 00182267889, 00182267901, 00182267989, 00223237501,

Generic name	National Drug Codes
	00223237502, 00223237601, 00223237602, 00223237701, 00223237702, 00223237801, 00223237802, 00223237901, 00223237902, 00302820001, 00302820201, 00302820401, 00302820601, 00302820801, 00304092101, 00304092201, 00304092301, 00304092401, 00306682480, 00339653712, 00339653812, 00339653912, 00339654012, 00339654112, 00339654212, 00339654312, 00339654412, 00339654512, 00349839001, 00349839100, 00349839101, 00349839201, 00359037910, 00359137910, 00359138010, 00359138110, 00359138210, 00359138310, 00364063901, 00364064001, 00364064002, 00364248601, 00378880101, 00378880110, 00378880201, 00378880210, 00378880301, 00378880310, 00378880401, 00378880410, 00378880501, 00378880510, 00378880601, 00378880610, 00378881001, 00378881010, 00378882501, 00378882510, 00378887501, 00378887510, 00403065330, 00403068301, 00403068330, 00403481701, 00405510601, 00405510701, 00406205201, 00406205210, 00406205301, 00406205310, 00406205401, 00406205410, 00406205501, 00406205510, 00406205601, 00406205610, 00406205701, 00406205801, 00406205901, 00406206401, 00406206410, 00527100301, 00527100310, 00527106401, 00527106410, 00527107201, 00527107210, 00536485101, 00536485110, 00536485201, 00536485210, 00536485301, 00536485310, 00536485401, 00536485501, 00555083102, 00555083105, 00555083202, 00555083205, 00555083302, 00555083305, 00555083402, 00555083405, 00555083502, 00555083504, 00555086902, 00555086905, 00555087402, 00555087405, 00555092502, 00555092602, 00580140601, 00580140701, 00580140801, 00580140901, 00580141001, 00590032435, 00590032496, 00615150929, 00615150953, 00615150963, 00615151029, 00615151053, 00615151063, 00615151229, 00615151253, 00615151263, 00615454729, 00615454753, 00615454763, 00615454829, 00615454853, 00615454863, 00615454929, 00615454953, 00615454963, 00615455029, 00615455129, 00615455729, 00615457729, 00677079301, 00677079401, 00677081301, 00677081401, 00677081501, 00719199210, 00719199310, 00719199313, 00719199410, 00719199510, 00725004401, 00725004410, 00725004501, 00725004510, 00725004601, 00725004610, 00725004701, 00725004710, 00725005001, 00725005010, 00779004401, 00779004501, 00781035207, 00781036307, 00781036407, 00781036607, 00781036907, 00781037707, 00781038107, 00781038607, 00781038707, 00814852214, 00832062500, 00832062513, 00832062600, 00832062613, 00832062700, 00832062710, 00832062713, 00832062725, 00832121100, 00832121101, 00832121110, 00832121189, 00832121200, 00832121201, 00832121210, 00832121289, 00832121300, 00832121301, 00832121310, 00832121389, 00832121400, 00832121401, 00832121410, 00832121489, 00832121500, 00832121501, 00832121510, 00832121589,

Generic name	National Drug Codes
	00832121600, 00832121601, 00832121610, 00832121689, 00832121700, 00832121701, 00832121710, 00832121789, 00832121800, 00832121801, 00832121850, 00832121889, 00832121900, 00832121901, 00832121950, 00832121989, 00839662606, 00839662616, 00839662706, 00839662716, 00839662916, 00839662806, 00839662816, 00839662906, 00839663006, 00839663016, 00904256060, 00904256160, 00904256260, 00904256270, 00904256280, 00904256360, 00904256460, 05167240323, 12071063601, 12071063701, 12071063710, 12071063801, 12280031230, 12280031260, 12280031290, 15330010001, 15330010010, 15330010101, 15330010110, 15330010201, 15330010210, 15330010601, 15330010701, 15330010801, 15330026601, 15330026701, 15330026801, 15330026810, 16590034030, 16590034060, 16590034090, 16590034130, 16590034160, 16590034190, 17236042401, 17236042410, 17236064201, 17236064701, 17236095901, 21695067230, 21695067330, 21695067360, 21695067430, 21695067530, 21695067730, 21695080130, 21695093930, 21695094030, 23490647801, 23490647802, 23490647803, 23490648001, 23490648002, 23490648003, 23490648101, 23490648102, 23490648103, 23490648201, 23490648202, 23490648203, 23490648301, 23490648302, 23490648303, 23490648401, 23490648402, 23490648403, 31722032701, 31722032710, 31722032801, 31722032810, 31722032901, 31722032910, 31722033001, 31722033010, 31722033101, 31722033110, 31722033201, 31722033210, 31722033301, 31722033401, 31722033501, 33261035500, 33261035507, 33261035514, 33261035520, 33261035521, 33261035528, 33261035530, 33261035560, 33261035590, 33261035607, 33261035614, 33261035620, 33261035621, 33261035628, 33261035630, 33261035660, 33261035690, 33261035707, 33261035710, 33261035714, 33261035720, 33261035721, 33261035728, 33261035730, 33261035760, 33261035790, 33261099000, 33261099030, 33261099060, 33261099090, 33261099730, 33261099760, 33261099790, 33261099830, 33261099860, 33261099890, 33261099930, 33261099960, 33261099990, 33358036000, 33358036130, 35356039730, 35356039760, 35356039790, 35356054090, 35356057130, 35356057160, 35356057190, 35356058230, 35356058260, 35356058290, 35356090630, 35356090690, 35470053301, 35470053401, 35470053501, 35470053509, 35470053601, 35470053701, 38779047404, 38779047405, 38779047410, 38779047425, 38779047503, 38779047504, 38779047505, 42549049630, 42549049730, 43063017614, 43063017630, 43063021830, 43063047130, 43063065530, 43353002130, 43353002135, 43353002140, 43353002145, 43353002150, 43353002153, 43353002155, 43353002160, 43353002161, 43353002170, 43353002311, 43353002315, 43353002320, 43353002321, 43353002325, 43353002328, 43353002330, 43353002335, 43353002338, 43353002340,

Generic name	National Drug Codes
	43353002344, 43353002345, 43353002346, 43353002347, 43353002350, 43353002353, 43353002355, 43353002357, 43353002359, 43353002360, 43353002361, 43353002365, 43353002368, 43353002370, 43353002378, 43353002830, 43353002860, 43353002930, 43353002935, 43353002940, 43353002945, 43353002953, 43353002960, 43353003030, 43353003060, 43353003330, 43353004930, 43353004960, 43353005011, 43353005015, 43353005020, 43353005021, 43353005025, 43353005028, 43353005030, 43353005035, 43353005038, 43353005040, 43353005044, 43353005045, 43353005046, 43353005047, 43353005050, 43353005053, 43353005055, 43353005057, 43353005059, 43353005060, 43353005061, 43353005065, 43353005068, 43353005070, 43353005078, 43353005330, 43353005430, 43353008930, 43353008960, 43353014230, 43353014235, 43353014240, 43353014245, 43353014253, 43353014260 43353049130, 43353049230, 43353049260, 43353049330, 43353049360, 43353049430, 43353057809, 43353057830, 43353057930, 43353058430, 43353058460, 43353058730, 47202250301, 47202266001, 47202266801, 47202272601, 47202272701, 47202273601, 49452813601, 49452813602, 49648092101, 49648092201, 49648092301, 49648092401, 49648092501, 49999009330, 49999041130, 49999057600, 49999057610, 49999057620, 49999057630, 49999057660, 49999057690, 49999082900, 49999092310, 49999092330, 49999092360, 49999092390, 50090002800, 50090203300, 50090214100, 50090258200, 50090258600, 50090258601, 50090261300, 50090261700, 50090264500, 50090264600, 50090264601, 50090265400, 50090293600, 50090293601, 50090293602, 50090311000, 50090311001, 50090312000, 50090326300, 50090500400, 50090505800, 50090539000, 50090543900, 50090543902, 50090544000, 50090546400, 50090546500, 51079090820, 51079090920, 51079091020, 51079091120, 51079091220, 51079091320, 51079091420, 51079091520, 51079091620, 51138005430, 51138005530, 51138005630, 51138005730, 51138005830, 51138005930, 51138006030, 51138006130, 51138006230, 51138017930, 51138018030, 51138018060, 51138018130, 51138018230, 51138018330, 51138018430, 51138018530, 51138018630, 51138018730, 51138019530, 51138019630, 51138019660, 51138019730, 51138019830, 51138019930, 51138020030, 51138020130, 51138020230, 51138020330, 51138048210, 51138048230, 51138048330, 51138048410, 51138048430, 51138048445, 51138048510, 51138048530, 51138048610, 51138048630, 51138048710, 51138048720, 51138048730, 51138048775, 51138048810, 51138048830, 51138048930, 51138049010, 51138049030, 51407034101, 51407034110, 51407034201, 51407034210, 51407034301, 51407034310, 51407034401, 51407034410, 51407034501, 51407034510, 51407034601, 51407034610, 51407034701, 51407034710,

Generic name	National Drug Codes
	51407034801, 51407034901, 51407078401, 51407078410, 51407078501, 51407078510, 51407078601, 51407078610, 51407078701, 51407078710, 51407078801, 51407078810, 51407078901, 51407078910, 51407079001, 51407079010, 51407079101, 51407079201, 51432090003, 51432090103, 51432090203, 51432090303, 51432090403, 51655027882, 51655028226, 51655028324, 51655028326, 51672402701, 51672402703, 51672402707, 51672402801, 51672402803, 51672402807, 51672402901, 51672402903, 51672402907, 51672403001, 51672403003, 51672403007, 51672403101, 51672403103, 51672403107, 51672403201, 51672403203, 51672403207, 51672403301, 51672403303, 51672403401, 51672403403, 51672403501, 51672403503, 51728054501, 51728054510, 51927247100, 52446060721, 52446060821, 52446060921, 52493061130, 52493061230, 52493061330, 52493069801, 52493069901, 52555000201, 52555000301, 52555000501, 52555000510, 52584004401, 52584004410, 52584004501, 52584004510, 52584005001, 52584005010, 52728013910, 52728014010, 52959092430, 52959092530, 52959092600, 52959092630, 53002104800, 53217000100, 53217000130, 53217000160, 53217000190, 53217001830, 53217001860, 53217001890, 53217021930, 53217021990, 53217023530, 53467017101, 53467017201, 53467017230, 54124017230, 54274009110, 54274009210, 54274009810, 54274009910, 54274010010, 54274010050, 54274023210, 54274023310, 54274023350, 54274023410, 54274023450, 54569015800, 54569015801, 54569015850, 54569015900, 54569015901, 54569015917, 54569015950, 54569021200, 54569021201, 54569021202, 54569042100, 54569187700, 54569187701, 54569257900, 54569444300, 54569444301, 54569493400, 54569493401, 54569493402, 54569586800, 54569586801, 54569586900, 54569622400, 54569622500, 54569622501, 54569631200, 54569631300, 54569631301, 54569639400, 54569642700, 54569854200, 54868082200, 54868082500, 54868121600, 54868125900, 54868125901, 54868125902, 54868125903, 54868125904, 54868125905, 54868125906, 54868125907, 54868212800, 54868212801 54868212802, 54868212803, 54868212900, 54868212901, 54868212902, 54868212903, 54868215400, 54868215401, 54868215402, 54868215403, 54868225200, 54868225201, 54868245400, 54868245401, 54868245402, 54868339900, 54868339901, 54868406300, 54868406301, 54868428600, 54868428601, 54868428602, 54868428603, 54868428604, 54868428605, 54868434900, 54868434901, 54868434902, 54868434903, 54868434904, 54868434905, 54868440000, 54868440001, 54868440002, 54868440003, 54868440004, 54868440200, 54868440201, 54868440202, 54868440203, 54868442200, 54868442201, 54868442202, 54868442203, 54868442204, 54868442205, 54868487100, 54868487101, 54868487102, 54868487103, 54868487300, 54868487301,

Generic name	National Drug Codes
	54868487302, 54868487303, 54868487304, 54868495000, 54868495001, 54868495002, 54868495003, 54868520700, 54868520701, 54868525500, 54868525501, 54868525800, 54868542500, 54977007330, 54977007399, 54977007430, 54977007499, 54977007530, 55045288000, 55045288001, 55045288008, 55045288100, 55045288108, 55045290200, 55045290208, 55048085630, 55048085730, 55048085830, 55048085930, 55081097600, 55084061201, 55084061301, 55153103901, 55154469500, 55154469600, 55154469700, 55154770100, 55154770200, 55154770300, 55154770400, 55154770600, 55154771600, 55175538003, 55175551003, 55289014397, 55289028601, 55289028630, 55289028650, 55289028697, 55289034030, 55289077314, 55289077330, 55289077360, 55289077390, 55700000530, 55700000560, 55700000590, 55829059110, 55829059210, 55829059310, 55887026430, 55887026460, 55887026482, 55887026490, 55887046430, 55887046460, 55887046490, 55887056730, 55887056760, 55887056790, 55887057710, 55887057730, 55887057760, 55887057790, 55887057810, 55887057830, 55887057860, 55887057886, 55887057890, 55887092690, 57237011901, 57237011999, 57237012001, 57237012099, 57237012101, 57237012199, 57237012201, 57237012299, 57237012301, 57237012399, 57237012401, 57237012499, 57237012501, 57237012599, 57237012601, 57237012699, 57237012701, 57237012799, 57362010811, 57362010813, 57362010819, 57362010884, 57362011011, 57362011013, 57362011084, 58016008300, 58016008330, 58016008360, 58016008390, 58016069700, 58016069730, 58016069760, 58016069790, 58517036030, 58864003014, 58864003030, 58864003530, 58864022314, 58864022330, 58864030114, 58864035715, 58864069814, 58864069830, 58864077315, 58864077330, 58864087930, 59772035204, 59772035207, 59772035208, 59772036304, 59772036307, 59772036308, 59772036404, 59772036407, 59772036408, 59772036607, 59772036907, 59772036908, 59772037704, 59772037707, 59772037708, 59772038107, 59772038607, 59772038707, 60346038125, 60346038130, 60346091830, 60429078401, 60429078410, 60429078415, 60429078430, 60429078445, 60429078477, 60429078501, 60429078510, 60429078515, 60429078530, 60429078535, 60429078540, 60429078545, 60429078560, 60429078577, 60429078590, 60429078601, 60429078610, 60429078615, 60429078630, 60429078645, 60429078677, 60429078701, 60429078710, 60429078715, 60429078730, 60429078745, 60429078777, 60429078801, 60429078810, 60429078815, 60429078830, 60429078845, 60429078877, 60429078901, 60429078910, 60429078915, 60429078920, 60429078925, 60429078930, 60429078935, 60429078940, 60429078945, 60429078950, 60429078960, 60429078975, 60429078977, 60429078990, 60429079001, 60429079010, 60429079015, 60429079030, 60429079045,

Generic name	National Drug Codes
	60429079077, 60429079101, 60429079115, 60429079130,
	60429079145, 60429079177, 60429079201, 60429079215,
	60429079230, 60429079245, 60429079277, 60760003130,
	60760003330, 60760003430, 60760004030, 60760004130,
	60760004330, 60760070630, 61919034130, 62584094477,
	62584098401, 62584098411, 62584098477, 62584098601,
	62584099401, 62584099411, 62584099477, 63187067410,
	63187067460, 63187067490, 63187074530, 63187075010
	63187075030, 63629133601, 63629254801, 63629254802,
	63629317701, 63629317702, 63629401701, 63629401702,
	63629401703, 63629401704, 63629401705, 63629412201,
	63629412202, 63629412203, 63629412204, 63629412205,
	63629412206, 63629441701, 63629441702, 63629441703,
	63629474801, 63629474802, 63629474803, 63629884301,
	63739036001, 63739036003, 63739036015, 63739036101,
	63739036103, 63739036110, 63739036115, 63739036201,
	63739036203, 63739036210, 63739036215, 63739036301,
	63739036303, 63739036310, 63739036315, 63739036401,
	63739036403, 63739036410, 63739036415, 65162076110,
	65162076111, 65162076210, 65162076211, 65162076310,
	65162076311, 65162076410, 65162076411, 65162076510,
	65162076511, 65162076610, 65162076611, 65162076710,
	65162076711, 65162076810, 65162076811, 65162076910,
	65162076911, 65243027403, 66105011010, 66105017070,
	66105017610, 66105017670, 66105051810, 66105051910,
	66105052110, 66105052310, 66116046930, 66116047030,
	66267026830, 66267028530, 66267062800, 66267062900,
	66267063000, 66267063100, 66267063200, 66267063300,
	66267063400, 66267063500, 66267063600, 66336024920,
	66336024930, 66336024960, 66336025020, 66336025030,
	66336025090, 66336025130, 66336025160, 66336025230,
	66336025260, 66336025290, 66336082530, 67544005215,
	67544005220, 67544005225, 67544005228, 67544005230,
	67544005235, 67544005238, 67544005240, 67544005245,
	67544005250, 67544005253, 67544005255, 67544005257,
	67544005260, 67544005261, 67544005265, 67544005268,
	67544005270, 67544005278, 67544007030, 67544019430,
	67544019530, 67544019540, 67544019545, 67544019553,
	67544019560, 67544031815, 67544031830, 67544031835,
	67544031840, 67544031845, 67544031850, 67544031853,
	67544031855, 67544031860, 67544031861, 67544031870,
	67544040115, 67544040130, 67544040135, 67544040140,
	67544040145, 67544040150, 67544040153, 67544040155,
	67544040160, 67544040161, 67544040170, 68084002701,
	68084002711, 68084002777, 68084014677, 68084014777,
	68084014877, 68115009230, 68115009330, 68115035930,
	68115035960, 68115035990, 68115039930, 68115052730,
	68115052760, 68115052790, 68115065900, 68258102601,
	68258102701, 68258606703, 68258606803, 68258607609,
	68258906401, 68258909701, 68258910101, 68258910201,

Generic name	National Drug Codes
	68258910401, 68382005201, 68382005210, 68382005301, 68382005310, 68382005401, 68382005410, 68382005501, 68382005510, 68382005601, 68382005610, 68382005616, 68382005701, 68382005801, 68382005901, 68382006401, 68382006410, 68788751501, 68788751503, 68788751506, 68788751509, 71335024301, 71335024302, 71335024303, 71335024304, 71335024305, 71335024306, 71335045201, 71335045202, 71335045203, 71335045204, 71335058001, 71610017330, 71610017335, 71610017340, 71610017345, 71610017930, 71610044811, 71610044815, 71610044820, 71610044821, 71610044825, 71610044828, 71610044830, 71610044835, 71610044838, 71610044840, 71610044844, 71610044845, 71610044846, 71610044847, 71610044850, 71610044853, 71610044855, 71610044859, 71610044860, 71610044861, 71610044868, 71610044878, 71610045830, 71610046230, 71610046235, 71610046240, 71610046245, 71610046253, 71610046260, 71610048030, 71610048035, 71610048040, 71610048045, 71610048053, 71610048060, 71610049030, 71610049130, 71610049815, 71610049820, 71610049825, 71610049830, 71610049835, 71610049840, 71610049845, 71610049850, 71610049853, 71610049855, 71610049860, 71610051930, 71610057430, 71610057435, 71610057440, 71610057445, 71610057453, 71610057460, 71610057515, 71610057520, 71610057525, 71610057530, 71610057535, 71610057538, 71610057540, 71610057545, 71610057550, 71610057553, 71610057555, 71610057560, 71610058930, 71610059330, 72189021071, 76282032701, 76282032710, 76282032801, 76282032810, 76282032901, 76282032910, 76282033001, 76282033010, 76282033101 76282033110, 76282033201, 76282033210, 76282033301, 76282033310, 76282033401, 76282033501

Table S2. ICD codes used to identify index bleeding events

Components	ICD-9-CM codes*	ICD-10-CM codes*
Gastrointestinal bleeding (any position and setting)	456.0, 456.20, 530.82, 531.0x, 531.2x, 531.4x, 531.6x, 532.0x, 532.2x, 532.4x, 532.6x, 533.0x, 533.2x, 533.4x, 533.6x, 534.0x, 534.2x, 534.4x, 534.6x, 535.01, 535.11, 535.21, 535.31, 535.41, 535.51, 535.61, 537.83, 562.02, 562.03, 562.12, 562.13, 568.81, 569.3, 569.85, 578.x, 44.43	I8501, I8511, K2211, K226, K250, K252, K254, K256, K260, K262, K264, K266, K270, K272, K274, K276, K280, K282, K284, K286, K2901, K2921, K2931, K2941, K2951, K2961, K2971, K2981, K2991, K31811, K3182, K5521, K5701, K5711, K5713, K5721, K5731, K5733, K5741, K5751, K5753, K5781, K5791, K5793, K625, K6381, K661, K920, K921, K922, K9161, K9162, K91840, K91841
Intracranial hemorrhage (any position and setting)	430, 431, 432.0, 432.1, 432.9, 852.0x, 852.2x, 852.4x, 853.0x	I6000, I6001, I6002, I6010, I6011, I6012, I602, I6030, I6031, I6032, I604, I6050, I6051, I6052, I606, I607, I608, I609, I610, I611, I612, I613, I614, I615, I616, I618, I619, I6200, I6201, I6202, I6203, I621, I629, S06340A, S06341A, S06342A, S06343A, S06344A, S06345A, S06346A, S06347A, S06348A, S06349A, S06350A, S06351A, S06352A, S06353A, S06354A, S06355A, S06356A, S06357A, S06358A, S06359A, S06360A, S06361A, S06362A, S06363A, S06364A, S06365A, S06366A, S06367A, S06368A, S06369A, S064X0A, S064X1A, S064X2A, S064X3A, S064X4A, S064X5A, S064X6A, S064X7A, S064X8A, S064X9A, S065X0A, S065X1A, S065X2A, S065X3A, S065X4A, S065X5A, S065X6A, S065X7A, S065X8A, S065X9A, S066X0A, S066X1A, S066X2A, S066X3A, S066X4A, S066X5A, S066X6A, S066X7A, S066X8A, S066X9A
Other bleeding (any position and setting)	285.1, 360.43, 362.43, 362.81, 363.61, 363.62, 363.72, 364.41, 372.72, 374.81, 376.32, 377.42, 379.23, 423.0x, 596.7x, 599.7x, 602.1x, 620.1, 621.4, 626.2, 626.5, 626.7, 626.8, 626.9, 719.1x, 782.7, 784.7, 784.8,	D62, D7801, D7802, D7821, D7822, E3601, E3602, E89810, E89811, G9731, G9732, G9751, G9752, H05231, H05232, H05233, H05239, H1130, H1131, H1132, H1133, H2100, H2101, H2102, H2103, H31301, H31302, H31303, H31309, H31311,

	786.3x, 958.2, 997.02, 998.11, Procedure code: 99.04	H31312, H31313, H31319, H31411, H31412, H31413, H31419, H3560, H3561, H3562, H3563, H35731, H35732, H35733, H35739, H4310, H4311, H4312, H4313, H44811, H44812, H44813, H44819, H47021, H47022, H47023, H47029, H59111, H59112, H59113, H59119, H59121, H59122, H59123, H59129, H59311, H59312, H59313, H59319, H59321, H59322, H59323, H59329, H9521, H9522, H9541, H9542, I312, I97410, I97411, I97418, I9742, I97610, I97611, I97618, I97620, J9561, J9562, J95830, J95831, L7601, L7602, L7621, L7622, M2500, M25011, M25012, M25019, M25021, M25022, M25029, M25031, M25032, M25039, M25041, M25042, M25049, M25051, M25052, M25059, M25061, M25062, M25069, M25071, M25072, M25073, M25074, M25075, M25076, M2508, M96810, M96811, M96830, M96831, N421, N857, N897, N920, N923, N930, N938, N939, N9961, N9962, N99820, N99821, R040, R041, R042, R0489, R049, R233, R310, R319, R58, T792XXA; Procedure codes: 30230N1, 30230P1, 30233N1, 30233P1, 30240N1, 30240P1, 30243N1, 30243P1, 30250N1, 30250P1, 30253N1, 30253P1, 30260N1, 30260P1, 30263N1, 30263P1

ICD, International Classification of Diseases; CM, Clinical Modification.

*Diagnosis codes, unless otherwise indicated.

Table S3. ICD codes used to identify stroke/SE and major bleeding outcomes

Composite outcome	Components	ICD-9-CM codes*	ICD-10-CM codes*
Stroke/SE	Hemorrhagic stroke	430.xx-432.xx†	I6000, I6001, I6002, I6010, I6011, I6012, I602, I6030, I6031, I6032, I604, I6050, I6051, I6052, I606, I607, I608, I609, I610, I611, I612, I613, I614, I615, I616, I618, I619‡
	Ischemic stroke	433.x1, 434.x1, 436	I6300, I63011, I63012, I63013, I63019, I6302, I63031, I63032, I63033, I63039, I6309, I6310, I63111, I63112, I63113, I63119, I6312, I63131, I63132, I63133, I63139, I6319, I6320, I63211, I63212, I63213, I63219, I6322, I63231, I63232, I63233, I63239, I6329, I6330, I63311, I63312, I63313, I63319, I63321, I63322, I63323, I63329, I63331, I63332, I63333, I63339, I63341, I63342, I63343, I63349, I6339, I6340, I63411, I63412, I63413, I63419, I63421, I63422, I63423, I63429, I63431, I63432, I63433, I63439, I63441, I63442, I63443, I63449, I6349, I6350, I63511, I63512, I63513, I63519, I63521, I63522, I63523, I63529, I63531, I63532, I63533, I63539, I63541, I63542, I63543, I63549, I6359, I636, I638, I639, I6789
	Systemic embolism	444.x, 445.x	I7401, I7409, I7410, I7411, I7419, I742, I743, I744, I745, I748, I749, I75011, I75012, I75013, I75019, I75021, I75022, I75023, I75029, I7581, I7589
Major Bleeding	Major gastrointestinal bleeding	456.0, 456.20, 530.82, 531.0x, 531.2x, 531.4x, 531.6x, 532.0x, 532.2x, 532.4x, 532.6x, 533.0x,	I8501, I8511, K2211, K226, K250, K252, K254, K256, K260, K262, K264, K266, K270, K272, K274, K276,

		533.2x, 533.4x, 533.6x, 534.0x, 534.2x, 534.4x, 534.6x, 535.01, 535.11, 535.21, 535.31, 535.41, 535.51, 535.61, 537.83, 562.02, 562.03, 562.12, 562.13, 568.81, 569.3, 569.85, 578.x, 44.43	K280, K282, K284, K286, K2901, K2921, K2931, K2941, K2951, K2961, K2971, K2981, K2991, K31811, K3182, K5521, K5701, K5711, K5713, K5721, K5731, K5733, K5741, K5751, K5753, K5781, K5791, K5793, K625, K6381, K661, K920, K921, K922, K9161, K9162, K91840, K91841
	Major intracranial hemorrhage	430, 431, 432.0, 432.1, 432.9, , 852.0x, 852.2x, 852.4x, 853.0x	I6000, I6001, I6002, I6010, I6011, I6012, I602, I6030, I6031, I6032, I604, I6050, I6051, I6052, I606, I607, I608, I609, I610, I611, I612, I613, I614, I615, I616, I618, I619, I6200, I6201, I6202, I6203, I621, I629, S06340A, S06341A, S06342A, S06343A, S06344A, S06345A, S06346A, S06347A, S06348A, S06349A, S06350A, S06351A, S06352A, S06353A, S06354A, S06355A, S06356A, S06357A, S06358A, S06359A, S06360A, S06361A, S06362A, S06363A, S06364A, S06365A, S06366A, S06367A, S06368A, S06369A, S064X0A, S064X1A, S064X2A, S064X3A, S064X4A, S064X5A, S064X6A, S064X7A, S064X8A, S064X9A, S065X0A, S065X1A, S065X2A, S065X3A, S065X4A, S065X5A, S065X6A, S065X7A, S065X8A, S065X9A, S066X0A, S066X1A, S066X2A, S066X3A, S066X4A, S066X5A, S066X6A, S066X7A, S066X8A, S066X9A

	Other major hemorrhage	285.1, 360.43, 362.43, 362.81, 363.61, 363.62, 363.72, 364.41, 372.72, 374.81, 376.32, 377.42, 379.23, 423.0x, 596.7x, 599.7x, 602.1x, 620.1, 621.4, 626.2, 626.5, 626.7, 626.8, 626.9, 719.1x, 782.7, 784.7, 784.8, 786.3x, 958.2, 997.02, 998.11<br>Procedure code: 99.04	D62, D7801, D7802, D7821, D7822, E3601, E3602, E89810, E89811, G9731, G9732, G9751, G9752, H05231, H05232, H05233, H05239, H1130, H1131, H1132, H1133, H2100, H2101, H2102, H2103, H31301, H31302, H31303, H31309, H31311, H31312, H31313, H31319, H31411, H31412, H31413, H31419, H3560, H3561, H3562, H3563, H35731, H35732, H35733, H35739, H4310, H4311, H4312, H4313, H44811, H44812, H44813, H44819, H47021, H47022, H47023, H47029, H59111, H59112, H59113, H59119, H59121, H59122, H59123, H59129, H59311, H59312, H59313, H59319, H59321, H59322, H59323, H59329, H9521, H9522, H9541, H9542, I312, I97410, I97411, I97418, I9742, I97610, I97611, I97618, I97620, J9561, J9562, J95830, J95831, L7601, L7602, L7621, L7622, M2500, M25011, M25012, M25019, M25021, M25022, M25029, M25031, M25032, M25039, M25041, M25042, M25049, M25051, M25052, M25059, M25061, M25062, M25069, M25071, M25072, M25073, M25074, M25075, M25076, M2508, M96810, M96811, M96830, M96831, N421, N857, N897, N920, N923, N930, N938, N939, N9961, N9962, N99820, N99821, R040, R041, R042, R0489, R049, R233, R310, R319, R58, T792XXA<br>Procedure codes: 30230N1, 30230P1, 30233N1, 30233P1, 30240N1, 30240P1, 30243N1,

	30243P1, 30250N1, 30250P1, 30253N1, 30253P1, 30260N1, 30260P1, 30263N1, 30263P1

ICD, International Classification of Diseases; CM, Clinical Modification.
*Diagnosis codes, unless otherwise indicated.
†To ensure bleeding was not due to trauma, events were excluded if any of the following ICD-9 codes were registered during a patient's hospitalization for hemorrhagic stroke: 800-804, 850-854.
‡To ensure bleeding was not due to trauma, events were excluded if any of the following ICD-10 codes were registered during a patient's hospitalization for hemorrhagic stroke: S0190XA, S020XXA, S020XXB, S02101A, S02101B, S02102A, S02102B, S02109A, S02109B, S02110A, S02110B, S02111A, S02111B, S02112A, S02112B, S02113A, S02113B, S02118A, S02118B, S02119A, S02119B, S0211AA, S0211AB, S0211BA, S0211BB, S0211CA, S0211CB, S0211DA, S0211DB, S0211EA, S0211EB, S0211FA, S0211FB, S0211GA, S0211GB, S0211HA, S0211HB, S0219XA, S0219XB, S022XXA, S022XXB, S0230XA, S0230XB, S0231XA, S0231XB, S0232XA, S0232XB, S02400A, S02400B, S02401A, S02401B, S02402A, S02402B, S0240AA, S0240AB, S0240BA, S0240BB, S0240CA, S0240CB, S0240DA, S0240DB, S0240EA, S0240EB, S0240FA, S0240FB, S02411A, S02411B, S02412A, S02412B, S02413A, S02413B, S0242XA, S0242XB, S02600A, S02600B, S02601A, S02601B, S02602A, S02602B, S02609A, S02609B, S02610A, S02610B, S02611A, S02611B, S02612A, S02612B, S02620A, S02620B, S02621A, S02621B, S02622A, S02622B, S02630A, S02630B, S02631A, S02631B, S02632A, S02632B, S02640A, S02640B, S02641A, S02641B, S02642A, S02642B, S02650A, S02650B, S02651A, S02651B, S02652A, S02652B, S0266XA, S0266XB, S02670A, S02670B, S02671A, S02671B, S02672A, S02672B, S0269XA, S0269XB, S0280XA, S0280XB, S0281XA, S0281XB, S0282XA, S0282XB, S0291XA, S0291XB, S0292XA, S0292XB, S060X0A, S060X1A, S060X9A, S061X0A, S061X1A, S061X2A, S061X3A, S061X4A, S061X5A, S061X6A, S061X7A, S061X8A, S061X9A, S062X0A, S062X1A, S062X2A, S062X3A, S062X4A, S062X5A, S062X6A, S062X7A, S062X8A, S062X9A, S06300A, S06301A, S06302A, S06303A, S06304A, S06305A, S06306A, S06307A, S06308A, S06309A, S06310A, S06311A, S06312A, S06313A, S06314A, S06315A, S06316A, S06317A, S06318A, S06319A, S06320A, S06321A, S06322A, S06323A, S06324A, S06325A, S06326A, S06327A, S06328A, S06329A, S06330A, S06331A, S06332A, S06333A, S06334A, S06335A, S06336A, S06337A, S06338A, S06339A, S06340A, S06341A, S06342A, S06343A, S06344A, S06345A, S06346A, S06347A, S06348A, S06349A, S06350A, S06351A, S06352A, S06353A, S06354A, S06355A, S06356A, S06357A, S06358A, S06359A, S06360A, S06361A, S06362A, S06363A, S06364A, S06365A, S06366A, S06367A, S06368A, S06369A, S06370A, S06371A, S06372A, S06373A, S06374A, S06375A, S06376A, S06377A, S06378A, S06379A, S06380A, S06381A, S06382A, S06383A, S06384A, S06385A, S06386A, S06387A, S06388A, S06389A, S064X0A, S064X1A, S064X2A, S064X3A, S064X4A, S064X5A, S064X6A, S064X7A, S064X8A, S064X9A, S065X0A, S065X1A, S065X2A, S065X3A, S065X4A, S065X5A, S065X6A, S065X7A, S065X8A, S065X9A, S066X0A, S066X1A, S066X2A, S066X3A, S066X4A, S066X5A, S066X6A, S066X7A, S066X8A, S066X9A, S06810A, S06811A, S06812A, S06813A, S06814A, S06815A, S06816A, S06817A, S06818A, S06819A, S06820A, S06821A, S06822A, S06823A, S06824A, S06825A, S06826A, S06827A, S06828A, S06829A, S06890A, S06891A, S06892A, S06893A, S06894A, S06895A, S06896A, S06897A, S06898A, S06899A, S069X0A, S069X1A, S069X2A, S069X3A, S069X4A, S069X5A, S069X6A, S069X7A, S069X8A, S069X9A.

Table S4. Pre-PSM baseline characteristics of apixaban and rivaroxaban initiators who had a bleeding event while on therapy

	Apixaban initiators (N=59,620)		Rivaroxaban initiators (N=21,004)	
Age				
Mean (SD)	76.16	8.78	74.21	9.50
Median (Q1, Q3)	77	71, 83	75	69, 82
Age category, n (%)				
18 to 54	1,085	1.82%	706	3.36%
55 to 64	3,935	6.60%	2,051	9.76%
65 to 74	17,140	28.75%	6,708	31.94%
75 to 79	12,423	20.84%	4,173	19.87%
≥80	25,037	41.99%	7,366	35.07%
Gender, n (%)				
Female	29,204	48.98%	9,321	44.38%
Male	30,407	51.00%	11,677	55.59%
Unknown	9	0.02%	6	0.03%
Site/type of index bleeding event, n (%)				
Gastrointestinal	13,058	21.90%	4,449	21.18%
Intracranial hemorrhage	3,045	5.11%	752	3.58%
Other	38,511	64.59%	13,884	66.10%
Multiple	5,006	8.40%	1,919	9.14%
Severity of index bleeding event, n (%)				
Major bleeding	3,582	6.01%	1,512	7.20%
Clinically relevant non-major bleeding	56,038	93.99%	19,492	92.80%
CCI score				
Mean (SD)	4.01	2.91	3.21	2.64
Median (Q1, Q3)	4	2, 6	3	1, 5
CHA_2DS_2-VASC score				
Mean (SD)	4.61	1.69	4.15	1.70
Median (Q1, Q3)	5	3, 6	4	3, 5
CHA_2DS_2-VASC score, n (%)				
0	203	0.34%	168	0.80%
1	1,294	2.17%	897	4.27%
2	4,599	7.71%	2,425	11.55%
3	9,296	15.59%	4,155	19.78%
≥4	44,228	74.18%	13,359	63.60%
HAS-BLED score				
Mean (SD)	3.95	1.10	3.66	1.09
Median (Q1, Q3)	4	3, 5	4	3, 4
HAS-BLED score (%)				
0	0	0.00%	0	0.00%
1	384	0.64%	295	1.40%
2	4,187	7.02%	2,354	11.21%
≥3	55,049	92.33%	18,355	87.39%
Baseline comorbidities, n (%)				
History of any bleeding	59,620	100.00%	21,004	100.00%
Congestive heart failure	24,905	41.77%	7,166	34.12%
Diabetes	24,058	40.35%	7,651	36.43%

Hypertension	54,220	90.94%	18,572	88.42%
Renal Disease	27,461	46.06%	6,974	33.20%
Liver Disease	6,064	10.17%	1,735	8.26%
Myocardial infarction	10,476	17.57%	2,912	13.86%
Dyspepsia or Stomach discomfort	13,229	22.19%	3,304	15.73%
Peripheral vascular disease	20,440	34.28%	6,106	29.07%
Transient ischemic attack	10,480	17.58%	2,601	12.38%
Alcoholism	1,998	3.35%	652	3.10%
Peripheral arterial disease	10,597	17.77%	3,432	16.34%
Coronary artery disease	30,202	50.66%	9,520	45.32%
Stroke/SE	9,530	15.98%	2,439	11.61%
All-cause hospitalization, n (%)	34,503	57.87%	10,265	48.87%
Days from DOAC initiation date to index bleeding event				
Mean (SD)	225.38	318.70	229.98	340.68
Median (Q1, Q3)	102	27, 289	98	27, 284
Index DOAC dose, n (%)				
Standard dose*	47,215	79.19%	15,481	73.71%
Low dose†	12,405	20.81%	5,523	26.29%

CCI, Charlson Comorbidity Index; CHA_2DS_2-VASc, Congestive heart failure, Hypertension, Age ≥75 years, Diabetes, Stroke, Vascular disease, Age 65–74 years, Sex category; DOAC, direct oral anticoagulant; HAS-BLED, Hypertension, Abnormal renal/liver function, Stroke, Bleeding history or predisposition, Labile international normalized ratio, Elderly, Drugs/alcohol; PSM, propensity score matching; Q, quartile; SD, standard deviation.

*5 mg for apixaban; 20 mg for rivaroxaban.

†2.5 mg for apixaban; 10 or 15 mg for rivaroxaban.

Table S5. Numbers of apixaban initiators censored for different reasons

	Major bleeding				Stroke/SE			
	Switchers (N=408)		Persistent users (N=2,040)		Switchers (N=408)		Persistent users (N=2,040)	
Reasons for censoring	N	%	N	%	N	%	N	%
Major bleeding outcome event	31	7.60	102	5.00	7	1.72	22	1.08
Death	35	8.58	182	8.92	39	9.56	196	9.61
Treatment switching	22	5.39	27	1.32	24	5.88	27	1.32
Treatment discontinuation	216	52.94	1138	55.78	229	56.13	1197	58.68
Study end	54	13.24	317	15.54	58	14.22	325	15.93
End of enrollment	50	12.25	274	13.43	51	12.50	273	13.38

SE, systemic embolism.

Table S6. Numbers of rivaroxaban initiators censored for different reasons

	Major bleeding				Stroke/SE			
	Switchers (N=901)		Persistent users (N=4,505)		Switchers (N=901)		Persistent users (N=4,505)	
Reasons for censoring	N	%	N	%	N	%	N	%
Stroke/SE outcome event	36	4.00	283	6.28	8	0.89	51	1.13
Death	70	7.77	334	7.41	73	8.10	354	7.86
Treatment switching	21	2.33	175	3.88	21	2.33	190	4.22
Treatment discontinuation	568	63.04	2531	56.18	590	65.48	2 696	59.84
Study end	100	11.10	462	10.26	104	11.54	477	10.59
End of enrollment	106	11.76	720	15.98	105	11.65	737	16.36

SE, systemic embolism.

Table S7. E-values for major bleeding and stroke/SE in apixaban and rivaroxaban initiators

Cohort	Outcome	E-value	E_{lower}*	E_{upper}†
Apixaban initiators	Major bleeding	2.230	1.399	-
	Stroke/SE	2.359	1.000	-
Rivaroxaban initiators	Major bleeding	2.029	-	1.310
	Stroke/SE	1.652	-	1.000

SE, systemic embolism.
*E_{lower} corresponds to the lower bound of the 95% confidence interval for the hazard ratio.
†E_{lower} corresponds to the upper bound of the 95% confidence interval for the hazard ratio.

Figure S1. Balance plots showing the standardized mean difference before and after propensity score matching for apixaban initiators (a) and rivaroxaban initiators (b). CCI, Charlson Comorbidity Index; DOAC, direct oral anticoagulant; SE, systemic embolism.

a

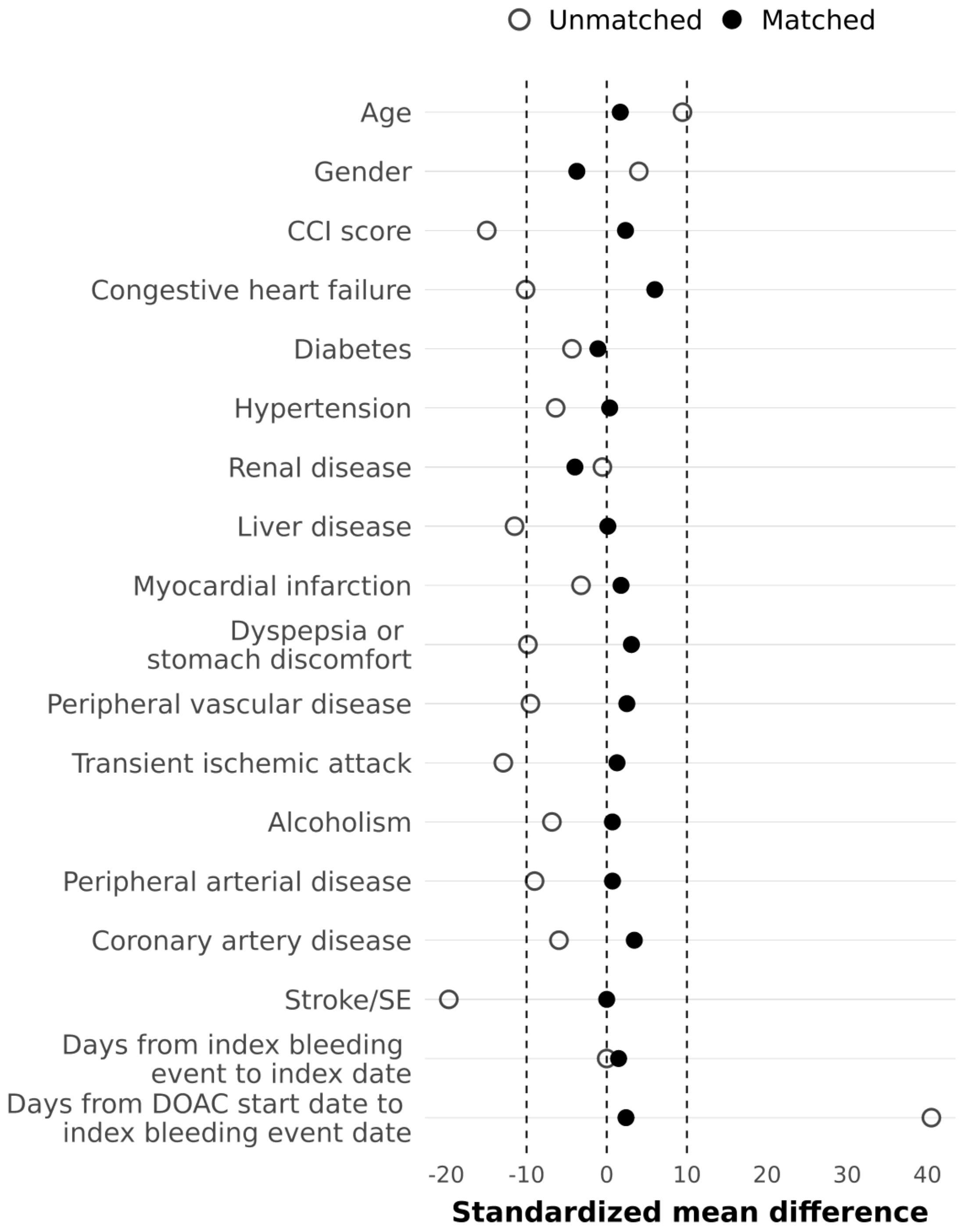

b

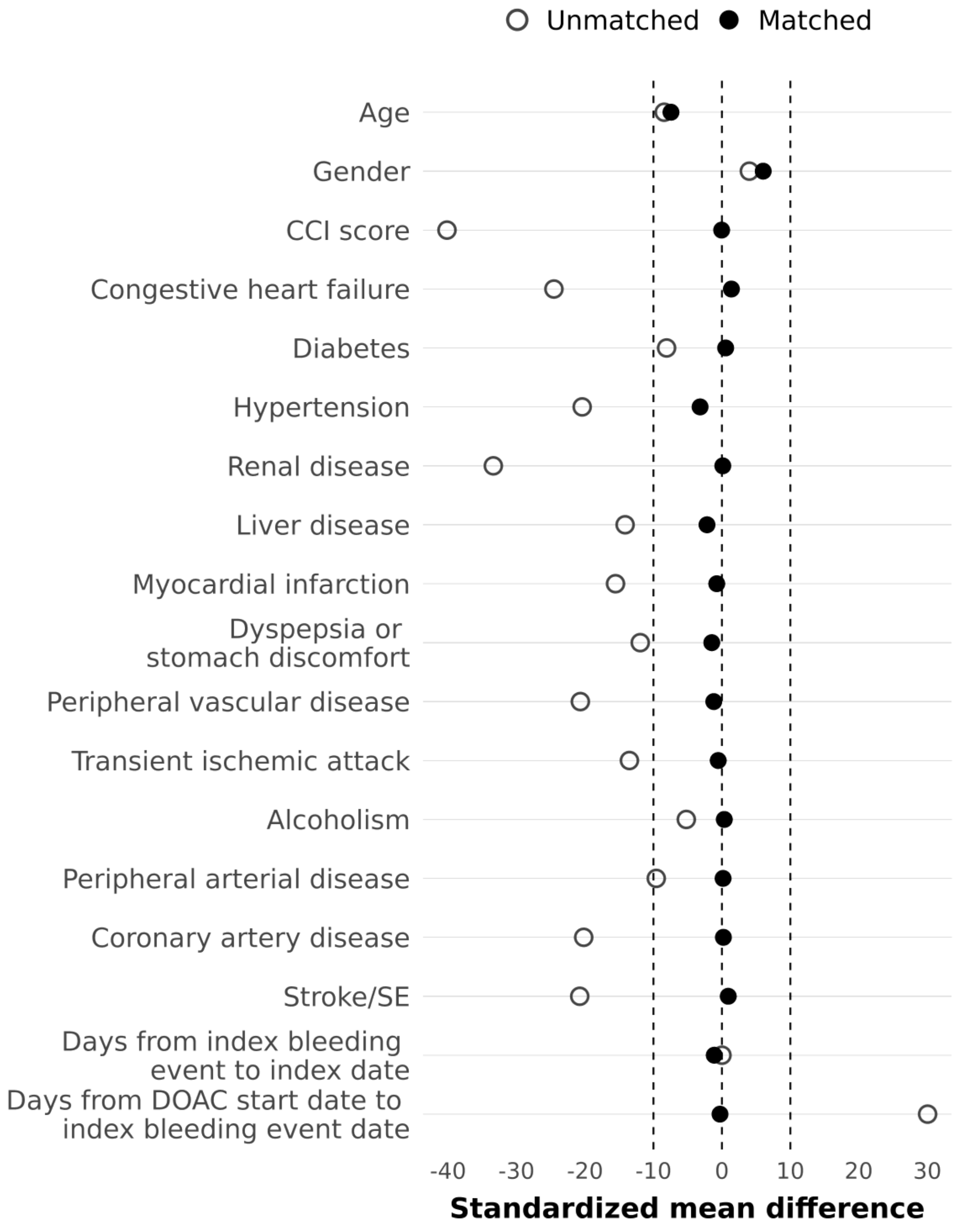

Unmatched
Matched
Age
Gender
CCI score
Congestive heart failure
Diabetes
Hypertension
Renal disease
Liver disease
Myocardial infarction
Dyspepsia or stomach discomfort
Peripheral vascular disease
Transient ischemic attack
Alcoholism
Peripheral arterial disease
Coronary artery disease
Stroke/SE
Days from index bleeding event to index date
Days from DOAC start date to index bleeding event date
-40
-30
-20
-10
0
10
20
30
Standardized mean difference

Figure S2. Schoenfeld residual plots for proportional-hazards assessment. (a) Major bleeding in apixaban initiators. (b) Major bleeding in rivaroxaban initiators. (c) Stroke/SE in apixaban initiators. (d) Stroke/SE in rivaroxaban initiators. Red dots represent Schoenfeld residuals at each event time. The solid black line shows the smoothed time-varying effect estimate, and dashed lines indicate its 95% confidence band. An approximately flat solid black line centered around zero suggests that the proportional-hazards assumption holds for that covariate. SE, systemic embolism.

a

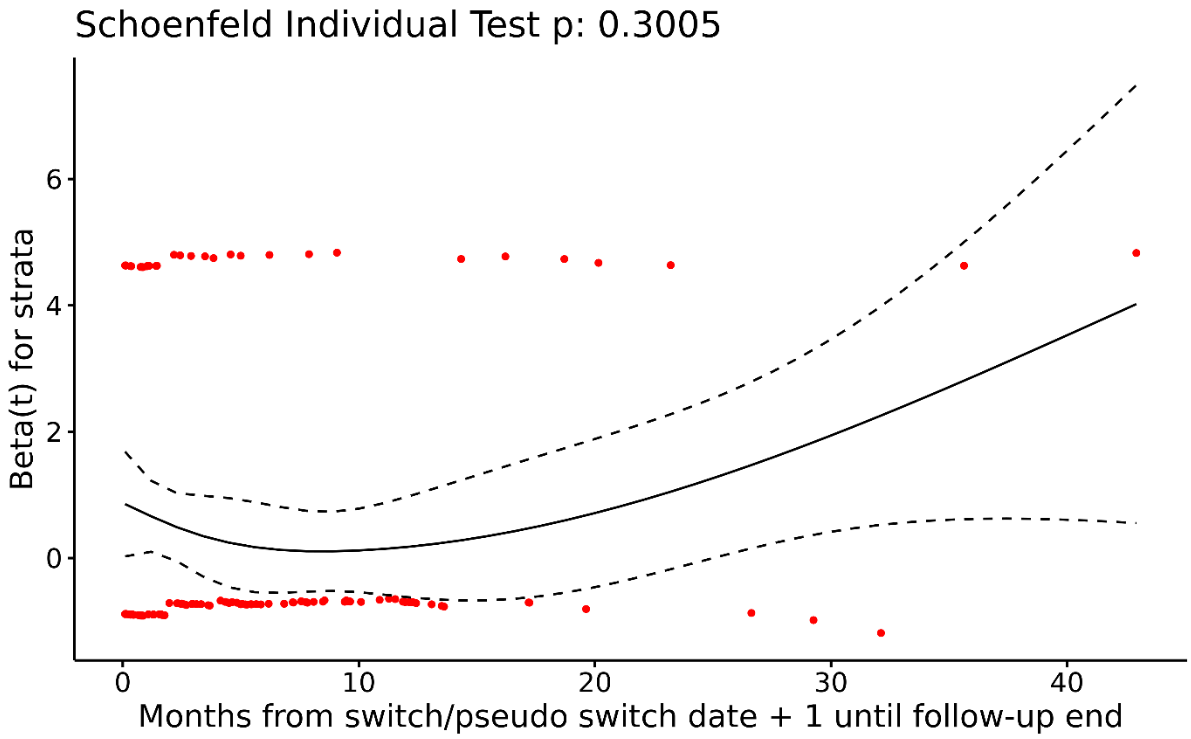

b

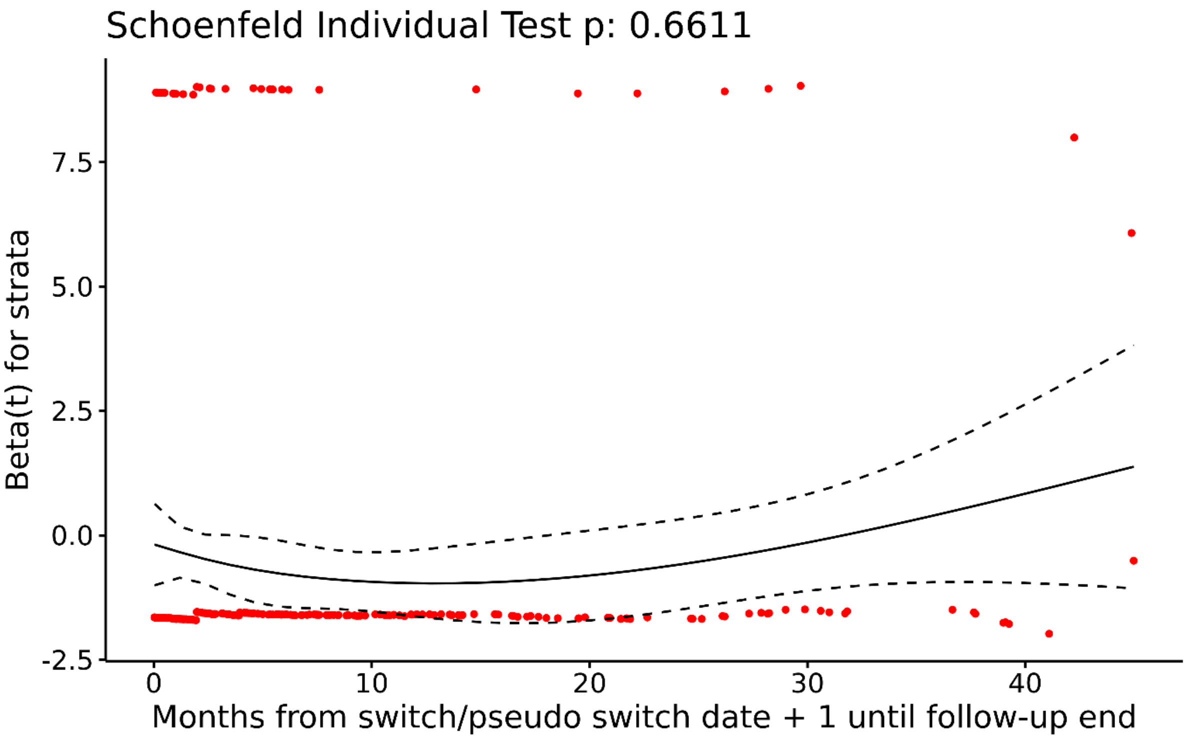
Schoenfeld Individual Test p: 0.6611
Beta(t) for strata
7.5
5.0
2.5
0.0
-2.5
0
10
20
30
40
Months from switch/pseudo switch date + 1 until follow-up end

c

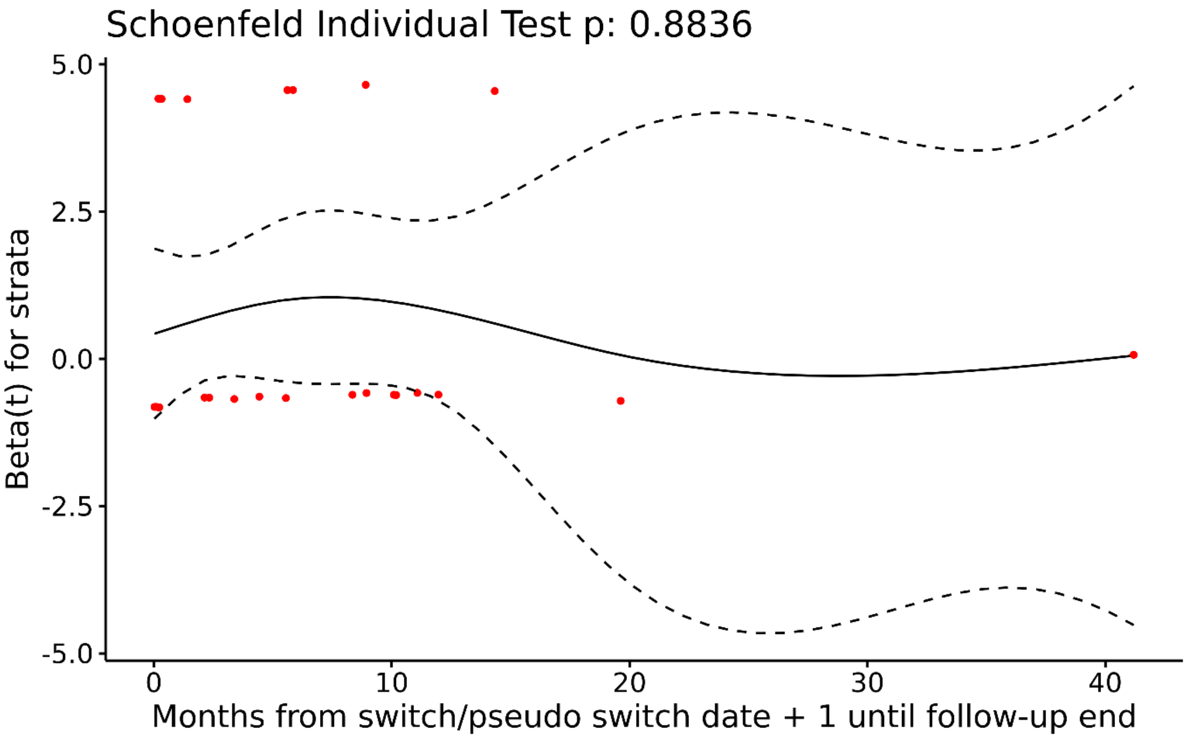
Schoenfeld Individual Test p: 0.8836
Beta(t) for strata
5.0
2.5
0.0
-2.5
-5.0
0
10
20
30
40
Months from switch/pseudo switch date + 1 until follow-up end

d

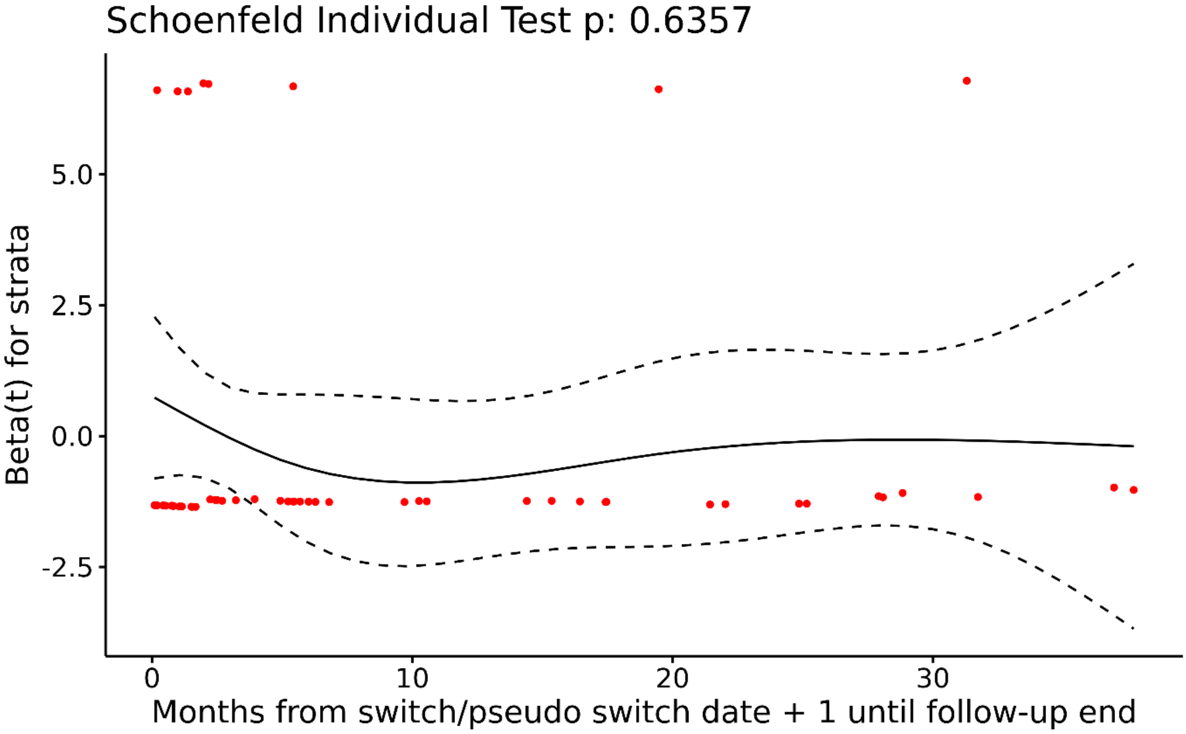
Schoenfeld Individual Test p: 0.6357
Beta(t) for strata
5.0
2.5
0.0
-2.5
0
10
20
30
Months from switch/pseudo switch date + 1 until follow-up end
